# CircFOXK2 promotes hepatocellular carcinoma progression and leads to a poor clinical prognosis via regulating the Warburg effect

**DOI:** 10.1186/s13046-023-02624-1

**Published:** 2023-03-15

**Authors:** Jun Zheng, Xijing Yan, Tongyu Lu, Wen Song, Yang Li, Jinliang Liang, Jiebin Zhang, Jianye Cai, Xin Sui, Jiaqi Xiao, Haitian Chen, Guihua Chen, Qi Zhang, Yubin Liu, Yang Yang, Kanghong Zheng, Zihao Pan

**Affiliations:** 1 Department of Hepatic Surgery and Liver Transplantation Center of the Third Affiliated Hospital of Sun Yat-Sen University, Organ Transplantation Research Center of Guangdong Province, Guangdong Province Engineering Laboratory for Transplantation Medicine, Guangzhou, 510630 China; 2 Guangdong Key Laboratory of Liver Disease Research, Key Laboratory of Liver Disease Biotherapy and Translational Medicine of Guangdong Higher Education Institutes, the Third Affiliated Hospital of Sun Yat-Sen University, Guangzhou, 510630 China; 3 Department of Anesthesiology, Sun Yat-Sen Memorial Hospital of Sun Yat-Sen University, Guangzhou, 510120 China; 4 Surgical ICU of the Third Affiliated Hospital of Sun Yat-Sen University, Guangzhou, 510630 China; 5 Department of General Surgery, Guangdong Provincial People’s Hospital (Guangdong Academy of Medical Sciences), Southern Medical University, Guangzhou, 510080 China; 6 Department of General Surgery of Guangdong Tongjiang Hospital, Foshan, 528300 China

**Keywords:** Circular RNA, Hepatocellular carcinoma, The Warburg effect, Encoding capacity, miRNA sponge

## Abstract

**Background:**

The Warburg effect is well-established to be essential for tumor progression and accounts for the poor clinical outcomes of hepatocellular carcinoma (HCC) patients. An increasing body of literature suggests that circular RNAs (circRNAs) are important regulators for HCC. However, few circRNAs involved in the Warburg effect of HCC have hitherto been investigated. Herein, we aimed to explore the contribution of circFOXK2 to glucose metabolism reprogramming in HCC.

**Methods:**

In the present study, different primers were designed to identify 14 circRNAs originating from the *FOXK2* gene, and their differential expression between HCC and adjacent liver tissues was screened. Ultimately, circFOXK2 (hsa_circ_0000817) was selected for further research. Next, the clinical significance of circFOXK2 was evaluated. We then assessed the pro-oncogenic activity of circFOXK2 and its impact on the Warburg effect in both HCC cell lines and animal xenografts. Finally, the molecular mechanisms of how circFOXK2 regulates the Warburg effect of HCC were explored.

**Results:**

CircFOXK2 was aberrantly upregulated in HCC tissues and positively correlated with poor clinical outcomes in patients that underwent radical hepatectomy. Silencing of circFOXK2 significantly suppressed HCC progression both in vitro and in vivo. Mechanistically, circFOXK2 upregulated the expression of protein FOXK2-142aa to promote LDHA phosphorylation and led to mitochondrial fission by regulating the miR-484/Fis1 pathway, ultimately activating the Warburg effect in HCC.

**Conclusions:**

CircFOXK2 is a prognostic biomarker of HCC that promotes the Warburg effect by promoting the expression of proteins and miRNA sponges that lead to tumor progression. Overall, circFOXK2 has huge prospects as a potential therapeutic target for patients with HCC.

**Supplementary Information:**

The online version contains supplementary material available at 10.1186/s13046-023-02624-1.

## Introduction

Current evidence suggests that hepatocellular carcinoma (HCC) is the third leading cause of cancer-related death worldwide [1, 2]. The past decades have witnessed unprecedented medical progress which has led to the advent of novel diagnostic and therapeutic approaches resulting in a significant reduction in the mortality rate of HCC. Nonetheless, according to the 2018 Global Cancer Statistics, 420, 000 new cases and 391, 000 000 liver cancer deaths have been reported in China [1]. The prognosis of HCC remains poor, with a 5-year overall survival (OS) of less than 25% [3]. Accordingly, over the past few years, there has been a burgeoning interest in exploring the molecular mechanisms underlying tumor progression and developing targeted treatments for this patient population.

In recent years, a significant number of noncoding RNAs (ncRNAs) have been identified to regulate the biological characteristics of HCC instead of side-products of splicing. Circular RNAs (circRNAs) represent a novel population of endogenous long ncRNAs (lncRNAs) that regulate gene expression and protein function in mammals, mainly composed of exons of the protein-coding genes and exhibit higher stability than the linear RNA owing to the circular structure to defy exonucleolytic RNA decay [4, 5]. There is a rich literature available substantiating the multiple regulatory functions of circRNAs, including microRNA (miRNA) sponging, interaction with proteins, and acting as protein translation templates, which may contribute to carcinogenesis and tumor progression [6–9]. In a previous study, we provided preliminary evidence that a novel protein (circFNDC3B-218aa) encoded by circFNDC3B could block colon cancer progression by regulating the Snail/FBP1 pathway [10]. Besides, several circRNAs have been associated with HCC. In this respect, circ-CDYL, circASAP1, circSLC3A2, circRHOT1, circMAT2B, and circMALAT1 have been documented as enhancers in HCC, while circMOT1, cSMARCA5 and circTRIM33-12 reportedly act as competing endogenous RNAs (ceRNAs) to suppress HCC progression [11–18]. However, the overall pathological effect and underlying mechanisms of circRNAs in HCC have been largely understudied, emphasizing the need for further research.

Metabolic reprogramming is a well-recognized hallmark of cancer cells. Intriguingly, despite the presence of oxygen, cancer cells produce energy via aerobic glycolysis rather than mitochondrial oxidative phosphorylation (OXPHOS), in addition to upregulated glucose uptake and lactate generation, this phenomenon is termed the Warburg effect [19, 20]. Based on the Warburg effect, adenosine triphosphates (ATP) and many molecules required for carcinogenesis are synthesized in HCC and other cancerous cells to regulate the tumor microenvironment, maintain cancer biology, and resist antitumor therapy [21]. In addition, recent studies have shown that FOXK2 is a critical transcription factor that regulates multiple biological processes and participates in the induction of aerobic glycolysis by strengthening the activity of pyruvate dehydrogenase kinase 1 and 4 to suppress the oxidation of pyruvate and to enhance the enzymatic machinery [22, 23]. Besides, it has been demonstrated that FOXK2 promotes the proliferation and migration of HCC cells mediated by activation of the PI3K/AKT signaling pathway [24]. However, whether the circRNA originated from the *FOXK2* gene is associated with the Warburg effect in HCC remains largely unclear.

In the present study, we identified a novel circRNA (circFOXK2, has_circ_0000817) that acted as a promoter in HCC and correlated with poor postoperative prognosis. Mechanistically, circFOXK2 exhibits protein-encoding (encodes the FOXK2-142aa protein to regulate the phosphorylation of LDHA) and miRNA sponging [binds to miR-484 to relieve silencing of mitochondrial adaptor fission 1 (Fis1)] roles to enhance the Warburg effect in HCC cells. In a nutshell, circFOXK2 has huge prospects as an effective prognostic biomarker and a potential target for HCC treatment.

## Methods and materials

### Clinical data and specimens

HCC and adjacent tumor-free liver tissues were obtained from ninety-two patients who underwent radical hepatectomy at the Department of Hepatic Surgery and Liver Transplantation Center of the Third Affiliated Hospital of Sun Yat-sen University (Guangzhou, China) between July 2014 and August 2018. All enrolled patients were histologically diagnosed without any history of other pre-operative treatment, including chemotherapy and radiotherapy. After being collected, the resected specimens were cut, snap-frozen in liquid nitrogen and subsequently stored at -80℃. This study was approved by the Ethics Committee of the Third Affiliated Hospital of Sun Yat-sen University, and the procedures were conducted abiding by the Declaration of Helsinki. All patients provided informed consent. Detailed information on the operated patients is provided in Supplemental Table 2.

### Cell lines and culture

The normal human hepatocyte line LO2 and four human HCC lines, Huh7, HepG2, Hep3B, and SK-Hep1, were purchased from the Chinese Academy of Sciences (Shanghai, China) and maintained in high glucose (4.5 g/L)-Dulbecco's Modified Eagle's medium (DMEM, Gibco, Life Technologies, Carlsbad, CA, USA) containing with 10% fetal bovine serum (FBS, PAN-Biotech, Germany). All cells were cultured at 37℃ under an atmosphere of 5% CO_2_. All cell lines were tested for mycoplasma and were mycoplasma-free when the experiments were conducted.

### Total RNA extraction and quantitative real-time polymerase chain reaction (qRT-PCR)

The primer sequences used in this study are listed in Supplemental Table 1. Total RNA extraction, reverse transcription and qRT-PCR were conducted as previously described [10]. The tissues were cut into 0.5 cm^3^ pieces and sequentially triturated in liquid nitrogen, while the cells were digested in 0.1% trypsin (Gibco, Life Technologies, Carlsbad, CA, USA). Then, total RNA extraction was performed using TRIzol (Invitrogen) according to the manufacturer's protocol. After confirming the amount and purity of total RNA using Biophotometer plus (Eppendorf, Germany), the Transcriptor First Strand cDNA Synthesis Kit (Roche Applied Science, USA) was used to reverse-transcribe total RNA to cDNA. cDNA was amplified using PCR Thermal Cycler (Bio-Rad, USA) by first heating at 65℃ for 10 min, incubating at 55℃ for 30 min, deactivating at 85℃ for 5 min and finally storage at 4℃ for 5 min. qRT-PCR was performed by SYBR Master Mix (Roche Applied Science) using a reverse transcription system (LC-480, Roche, USA). Glyceraldehyde-3-phosphate dehydrogenase (GAPDH) was used to normalize gene expression data.

### RNA fluorescence in situ hybridization

RNA fluorescence in situ hybridization (RNA-FISH) was conducted to visualize the location of circFOXK2 (RiboBio, Guangzhou, China) and miR-484 (BersinBio, Guangzhou, China) in cells using Fluorescent in Situ Hybridization Kits according to the manufacturer's instructions. The cells were treated with probes targeting circFOXK2 (hsa_circ_0000817) and miR-484 at 4℃ overnight and observed under a Zeiss 880 confocal microscope (Nikon Instruments, Melville, New York, United States) after being stained with DAPI for 2 min.

### Cell proliferation assay

The proliferation potential of HCC cells was detected using a cell counting kit-8 (CCK8, KeyGEN BioTECH, Jiangsu, China) according to the manufacturer's protocol. After being treated according to the predefined group design, the cells were seeded in a 96-well plate at a density of 4,000 cells per well and cultured for 24, 48, 72, 96 and 120 h. The cell viability of each group was determined by the optical density (OD) values at 450 nm using a microplate reader (Tecan Spark 10 M, Austria).

### Colony formation assay

The colony formation assay was used to evaluate the cloning ability of HCC cells. HCC cells in each group were seeded in 6-well plates at a density of 1,000 cells/well. Then, the cells were cultured in an incubator for 2 weeks. The colonies were visualized and counted after being fixed with 4% paraformaldehyde (PFA) and stained with crystal violet.

### Flow cytometry analysis

To measure cell apoptosis, a PI/Annexin V-FITC Apoptosis Detection Kit (BD, USA) was used according to the manufacturer's instructions. Briefly, 1 × 10^6^ HCC cells in each group were collected and rinsed twice with a washing buffer. Subsequently, the cells were incubated with Annexin V and PI for 15 min in the dark at room temperature. The cell apoptosis rate was determined using flow cytometry (BD Biosciences, San Jose, CA, USA).

### Transwell invasion assay

2 × 10^5^ HCC cells were resuspended in serum-free DMEM medium and seeded into the upper chamber of Transwell plates with 8.0-μm pores (Corning Costar, Corning, NY, USA). DMEM medium containing 10% FBS was added into the lower chamber. The cells were maintained in an incubator at 37℃ for 24 h. Then, the non-migrated cells on the upper compartment were removed. Subsequently, the cells were fixed with 4% PFA for 30 min and stained with crystal violet for 15 min. Images were captured under an inverted light microscope (Leica, German) and counted using ImageJ software (National Institutes of Health, USA).

### Wound healing assay

The cells in each group were seeded in 6-well plates for 24 h. A straight scratch was created using the tip of a sterilized pipette. Then, cells were washed gently with D-Hanks and cultured in DMEM containing 1% FBS. The samples were detected and photographed at 0 and 24 h under an inverted light microscope (Leica).

### Western blot assay

The cells were lysed in cold radioimmunoprecipitation assay (RIPA) Lysis Buffer containing Tris–HCL (pH 7.4, 50 mM), NaCl (150 mM), 0.1% Triton (100 ×), sodium dodecyl sulfate (SDS, 10%), sodium deoxycholate (10%), ethylene diamine tetraacetic acid (EDTA, 2 mM) and protease cocktail inhibitor (KeyGEN BioTECH, Jiangsu, China) for 20 min on ice. After total protein quantitation using a bicinchoninic protein assay (KeyGEN BioTECH), 30 μg proteins were electrophoresed using 12% SDS polyacrylamide gel electrophoresis (PAGE) and subsequently transferred onto polyvinylidene difluoride membranes (PVDF, Millipore, Billerica, MA, USA). 5% non-fat milk was used to block the non-specific antigen of the membranes for 1 h at room temperature. Then, the membranes were incubated with the corresponding primary antibodies at 4℃ overnight. After washing three times, the membranes were treated with secondary antibodies (anti-rabbit IgG, 1:5000, Sigma-Aldrich) for 1 h at room temperature. The blots were visualized by a ChemiDoc™ MP Imaging System (Bio-Rad, CA, USA) after treatment with an enhanced chemiluminescence (ECL) substrate. The intensities of the blots were evaluated using ImageJ software.

### Metabolism assay (including lactate, pyruvate, and ATP assays)

1. Lactate: Lactate concentration in the cell culture supernatant was determined utilizing a Lactate Assay Kit (BioVision) following the manufacturer's instructions. In brief, after each group was treated as previously defined during the study design, the supernatant was harvested and centrifuged for 10 min to remove the insoluble portion. The samples were incubated with a Lactate Assay Kit for 30 min in the dark and detected by a microplate reader (Tecan Spark 10 M, Austria) at a wavelength of 570 nm.
2. Pyruvate activity: For determination of pyruvate activity, the cells in each group were collected and extracted in the Pyruvate Assay Buffer (4 volume, BioVision). After centrifugation for 10 min to remove insoluble material, the supernatant was detected using a Pyruvate Colorimetric Assay Kit (BioVision). The reaction mixture was assayed at a wavelength of 570 nm using a microplate reader after incubation at room temperature in the dark for 30 min.
3. Intracellular ATP: Intracellular ATP was measured by an ENLITEN ATP Assay System Bioluminescence Detection Kit (Promega, USA) following the manufacturer's instructions. After each group was treated as previously defined during the study design and cultured in high-glucose DMEM containing 10% FBS for 48 h, the cells were washed with PBS three times and subsequently extracted in the ATP Assay Buffer (Promega). The ENLITEN ATP Assay System Bioluminescence Detection Kit was mixed with the supernatant, followed by incubation in the dark for 30 min at room temperature. The OD values at 570 nm were detected using a microplate reader.

### The NADH/NAD + assays

The NADH/NAD + Quantification Colorimetric Kit (BioVision) was used to determine the NADH/NAD + ratio of HCC cells. All procedures were conducted following the manufacturer's protocol.

### Extracellular acidification rate (ECAR) and oxygen consumption rate (OCR) quantification

The extracellular acidification rate and oxygen consumption rate were detected using the Seahorse XF^e^ 96 Extracellular Flux Analyzer (Seahorse Bioscience) following the manufacturer's instructions. The results were analyzed using the Seahorse XF-96 Wave software. The procedures for these two assays were as follows:

- ECAR: The cells from each group were seeded in a 24-well cell culture XF microplate (Seahorse Bioscience) at a density of 25,000 cells/well. After being cultured overnight to allow cell adherence, the cells were washed twice with assay medium and subsequently incubated with assay medium [the DMEM containing L-glutamine (2 mM), pH 7.4] in a CO_2_-free incubator at 37℃ for 1 h. In addition, 80 mM glucose, 9 μM oligomycin and 1 M 2-DG were loaded to cartridge ports A, B, and C. All values were normalized according to the cell number through the crystal violet assay. ECAR was identified as glycolysis rate after being treated with glucose, and the glycolytic capacity was determined after oligomycin treatment.
- OCR: ATP synthesis was inhibited by oligomycin (1 μM, Sigma), then maximal OCR was measured by adding trifluoromethoxy carbonyl cyanide phenylhydrazone (FCCP, 500 nM, Sigma), and mitochondrial respiration was inhibited using antimycin A (the mitochondrial complex I inhibitor rotenone + the mitochondrial complex III inhibitor, 1 μM, Sigma). The values were also normalized according to the cell number.

### Detection of intracellular mitochondrial morphology

The mitochondrial morphology in the HCC cells was observed by staining with a MitoTracker Green FM probe (Invitrogen) in the dark for 30 min at 37℃. Images were obtained under a Zeiss 880 confocal microscope to assess the number and morphology of mitochondria. Finally, the percentage of cells with small and round mitochondria was calculated.

### Co-immunoprecipitation

The cells were lysed in 4℃ cold co-IP buffer [HEPES (10 mM, pH 8.0), NaCl (300 mM), EDTA (0.1 mM), NP-40 (0.2%), glycerol (20%), protease and phosphatase inhibitors] for 30 min. The lysates were centrifuged at 10,000 g for 5 min and cleared after being treated with protein A/G agarose (Gibco BRL, Grand Island, NY, USA) for 15 min at 4℃. The pre-cleared supernatant was stirred in a horizontal shaker and incubated with the indicated primary antibodies at 4℃ overnight. Next, the protein complexes were harvested and incubated with the protein G beads for 120 min at 4℃. Finally, the samples were separated via SDS-PAGE.

### Mass spectrometry analysis by LC–MS/MS

Mass spectrometry analysis was conducted as previously described [10]. In brief, total proteins were first separated by SDS-PAGE (12%). Then, the protein bands near 15 kDa were manually cut according to the size and digested by sequencing-grade trypsin (Promega, Madison, WI, USA). Peptide mixtures were extracted, dried, and finally loaded to LC–MS/MS (Thermo Fisher Scientific, Waltham, MA, USA) for detecting the protein sequences. The National Center for Biotechnology Information nonredundant protein database with Mascot (Matrix Science, Boston, MA, USA) was used to analyze the fragment spectra.

### RNA immunoprecipitation (RIP)

After cross-linking with 1% formaldehyde in ice-cold PBS for 10 min, the cells were collected and lysed in RIP lysis buffer, followed by treatment with Dynabeads protein G conjugated with anti-AGO2 or anti-IgG and rotating overnight at 4℃. The immunoprecipitated RNAs were obtained using TRIzol reagent and measured by RT-qPCR with specific primers.

### Dual-luciferase reporter assay

The sequences of circFOXK2 with mutated miR-484 binding sites or wild-type were established and inserted into luciferase vectors. Then, these vectors and miR-484 mimics were co-transfected into HEK-293 T cells for 48 h. The luciferase activity was detected by a dual-luciferase reporter assay system (Promega, USA) following the manufacturer's instructions. The Renilla luciferase internal control was used to normalize the results.

### Biotin-labeled miRNA capture

The RNA pull-down assay was carried out as previously documented [25]. The biotin-labeled miR-484 mimic (GenePharma, China) was transfected into the stably overexpressing circFOXK2 HCC cells for 48 h. After being washed and blocked by yeast tRNA for 2 h at 4℃, the streptavidin-Dyna beads M-280 were incubated with the cell lysates overnight at 4℃ to pull down the biotin-coupled RNA complex. The abundance of circFOXK2 in bound sections was detected by qRT-PCR.

### Animals

Five-week-old male BALB/c nude mice were purchased from the Biomedical Research Institute of Nanjing University (Jiangsu, China) and housed under conditions following the Guidelines of Sun Yat-sen University for Animal Experimentation. All mice were housed under specific pathogen-free (SPF) conditions at the Laboratory Animal Center of Sun Yat-sen University with 12 h dark/light cycle, 50% humidity, 20℃ temperature and were fed standard laboratory diet and water ad libitum.

### Animal xenograft experiments

All in vivo experiments were approved by the Ethics Committee of Sun Yat-sen University (Guangzhou, China) and complied with the institutional guidelines. The xenograft model was established as previously described [26, 27]. 5 × 10^6^ HCC cells (Huh7) were suspended in Matrigel and injected subcutaneously into the left back of the mice. The length and width of the tumors were measured every three days when the tumors became palpable. Four weeks after implantation, the tumors were harvested for further analysis after sacrificing the mice. The weight and volume of samples were measured. The formula for calculating the tumor volume was as follows: tumor volume = (tumor length × tumor width^2^)/2. Moreover, lung metastasis models were established to detect the invasion potential of HCC cells in vivo. In brief, an empty vector, a sh-circFOXK2, or sh-circFOXK2 + a FOXK2-142aa overexpression plasmid, was transfected to the GFP-labeled HCC cells followed by intravenous injection through the tail vein at a density of 2 × 10^6^ cells/mouse. The mice were sacrificed after four weeks, and the lungs were visualized and photographed using the IVIS@ Lumina II system.

### Immunohistochemistry staining

The protocol of immunohistochemistry staining was conducted as previously described [28]. In brief, 4 μm-thick paraffin-embedded sections were dewaxed and dehydrated, followed by incubation with H_2_O_2_ (3%) for 10 min at 37℃. For antigen repair, the sections were treated with EDTA (pH 8.0) at 95℃ for 20 min and then cooled down to 25℃. After blocking the non-specific antigens with normal goat serum, the sections were incubated with primary antibodies overnight at 4℃ and the secondary antibody for 30 min at 37℃. Finally, the sections were treated with diaminobenzidine and detected under a light microscope (Leica, Germany).

### DNA/RNA transfection

The control plasmid (vector control), circFOXK2 overexpressing vector, circFOXK2-flag vector, circFOXK2-mut vector, circFOXK2-flag-mut vector and FOXK2-142aa vector were obtained from General Biosystems (Anhui, China). ShRNA knocking down circFOXK2, siRNA targeting LDHA and miR-484 mimics or inhibitors were purchased from GenePharma (GenePharma Corporation, Shanghai, China). After reaching approximately 60% confluence, the vectors were transfected to the cells using a Lipofectamine® 3000 transfection kit (Invitrogen, Carlsbad, CA, USA), while the shRNA, siRNA and miRNA mimics or inhibitors were transfected using Lipofectamine RNAiMax (Invitrogen) according to the manufacturer's protocol. The efficiency of transfection was determined by qRT-PCR.

### Statistical analysis

Statistical analyses were performed by GraphPad Prism (version 5.0, USA) and SPSS 23.0 (IBM, SPSS, Chicago, IL., USA). The relationship between circFOXK2 expression and the clinicopathological characterizations of HCC was analyzed by the Chi-square test. In addition, univariate and multivariate analyses were performed using a Cox proportional hazards regression model to assess the clinical value of circFOXK2. Survival analysis was carried out by the Kaplan–Meier method, and differences in overall survival were analyzed using the log-rank test. All in vitro experiments were independently repeated three times. As appropriate, the significant difference was assessed by the Mann–Whitney U test, Wilcoxon rank-sum test or unpaired two-tailed Student's t-test. A probability (*p*) value less than 0.05 was statistically significant. *p < 0.05, **p < 0.01, ***p < 0.001.

## Results

### Characterization of circFOXK2 in HCC

**Fig. 1:**
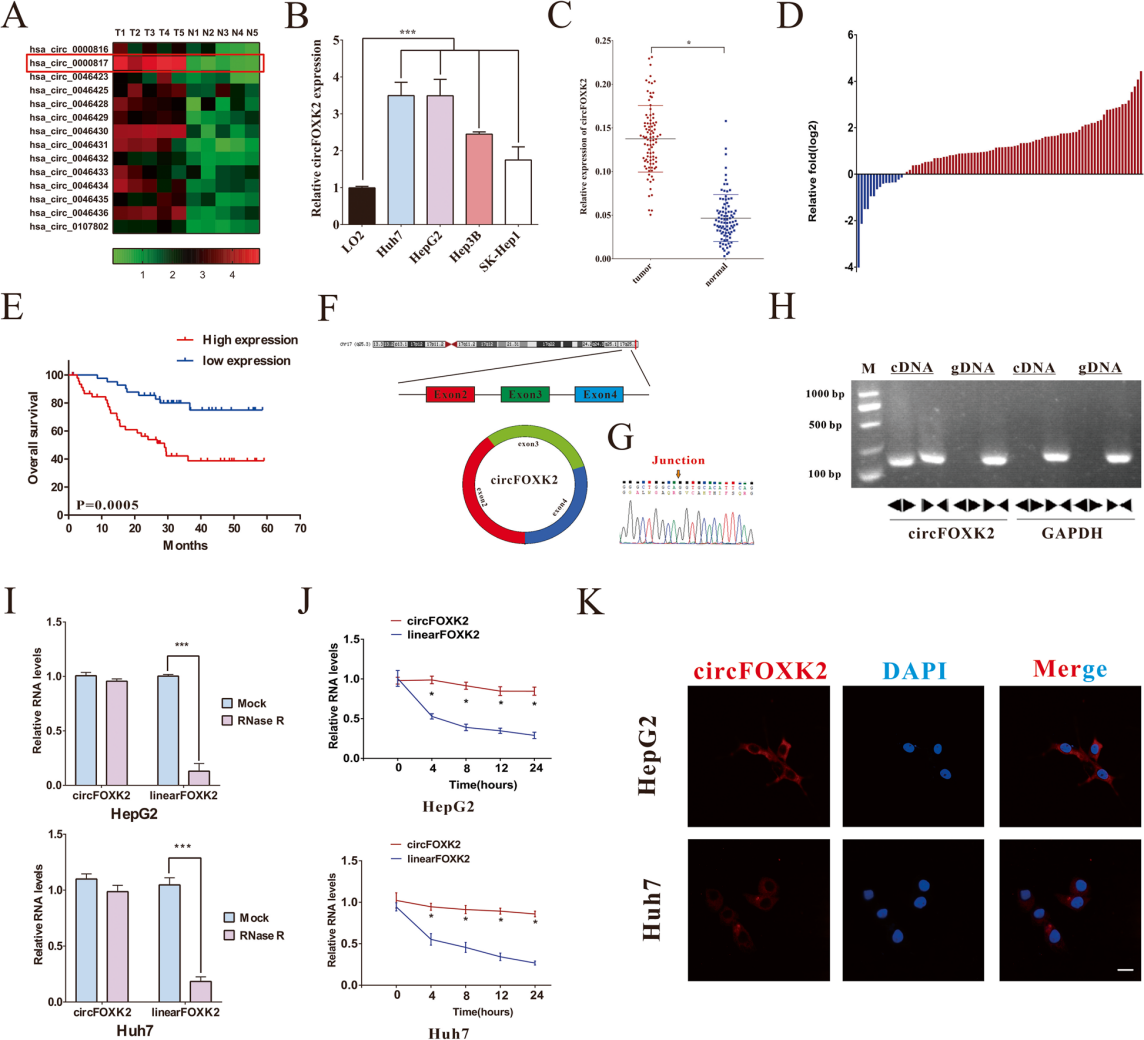
The identification and characterizations of circFOXK2 in HCC. **A** Heatmap exhibiting FOXK2 gene-derived circRNAs in HCC tissue compared with ANTs detected by qRT-PCR. **B** The expression of circFOXK2 in human HCC cell lines (Huh7, HepG2, Hep3B, and SK-Hep1) and normal hepatocyte line (LO2) were detected by RT-qPCR. **C** qRT-PCR was used to confirm the expression of circFOXK2 between 92 pairs of human HCC tissues and their counterpart adjacent normal tissue. **D** The expression of circFOXK2 was significantly high in 84% of HCC patients. **E** Kaplan–Meier's analyses of the OS of 92 HCC patients with high and low expression of circFOXK2. **F** Schematic illustration exhibiting the circularization of three exons, including exon2, exon3 and exon4, forming circFOXK2. **G** Sanger sequencing was used to demonstrate the back-splicing junction of circFOXK2. The arrow represents the special splicing junction of circFOXK2. **H** Through PCR analysis, divergent primer detected circular RNAs in cDNA but not in gDNA. **I** qRT-PCR was conducted to measure the relative level of circFOXK2 and FOXK2 linear RNA treated with or without RNase R. **J** The relative level of circFOXK2 and FOXK2 linear RNA in different time points was determined by qRT-PCR. **K** Localization of circFOXK2 in HepG2 and Huh7 cells was visualized by FISH. circFOXK2 probes were tagged with Cy3 (red) and DAPI (blue) was identified as nuclei. Data were represented as means ± SD with at least three independent experiments. *p < 0.05, **p < 0.01, ***p < 0.001

It has been reported that FOXK2 can yield heterogeneous effects in different types of cancer and may possess various biological functions [22, 23]. In addition, overwhelming evidence substantiates that circRNAs are generated from precursor mRNAs, and some participate in their host gene functions. In the present study, we first quantified the expressions of 14 circRNAs from FOXK2 in our samples. The results revealed that hsa_circ_0000817 (circFOXK2) was significantly increased in the HCC tissues compared to their adjacent liver tissues (Fig. 1A). Moreover, the expression of hsa_circ_0000817 was higher in multiple HCC cell lines (Huh7, HepG2, Hep3B, and SK-Hep1) than in the normal hepatocyte cell line (LO2) (Fig. 1B). We also performed qRT-PCR assays to investigate circFOXK2 expression in ninety-two pairs of clinical specimens from our center and further validated that compared with the counterpart adjacent liver tissues, circFOXK2 was significantly upregulated in the HCC tissues (Fig. 1C), with 84% (77 of 92) of HCC tissues presenting high cirFOXK2 expression (Fig. 1D). To assess the correlation between circFOXK2 expression and clinicopathological features/prognostic outcomes of HCC patients, we classified the HCC patients into high and low circFOXK2 expression groups according to the median circFOXK2 expression value. We found no significant correlation between circFOXK2 expression and the clinicopathological characteristics, indicating taht circFOXK2 was an independent prognostic factor for HCC after radical hepatectomy (Supplemental Table 2). Kaplan–Meier analysis showed that high expression of circFOXK2 was significantly associated with poorer OS compared to the low expression group (Fig. 1E). Next, the characterization of circFOXK2 was conducted. Our bioinformatics analysis showed that circFOXK2 (hsa_circ_0000817) is an exonic circRNA composed of the 2^nd^, 3^rd^, and 4^th^ exon of the *FOXK2* gene with a length of 490 nucleotides (Fig. 1F). Sanger sequencing demonstrated the back-splicing junction site of circFOXK2, consistent with the annotation from the circRNA website (www.cirbase.com) (Fig. 1G). In addition, we designed two kinds of primers and conducted agarose gel electrophoresis for detecting reverse-transcribed DNA (cDNA) and genomic DNA (gDNA). As expected, we found that circFOXK2 could amplify from cDNA but not from the different primers of gDNA (Fig. 1H). Previous studies demonstrated that linear RNA had a 3' to 5' exoribonuclease, which was conveniently digested by RNase R, while circRNA did not due to it did not contain a poly-A tail [29]. Accordingly, RNase R was used before reverse transcription experiments to further verify the circular characterizations of circFOXK2. Our results corroborated that unlike the linear form of FOXK2, circFOXK2 was resistant to RNase R treatment (Fig. 1I). Moreover, the half-life of circFOXK2 and FOXK2 linear RNA was detected using actinomycin D to block their transcription. It was found that circFOXK2 was more stable than FOXK2 linear RNA (Fig. 1J). The FISH assay for HepG2 and Huh7 cells confirmed that circFOXK2 was mainly expressed in the cytoplasm of HCC cells (Fig. 1K). Taken together, these results suggest that the expression of circFOXK2 (hsa_circ_0000817) in HCC tissues is high and correlates with a poor prognosis.

### CircFOXK2 enhances tumor progression and the Warburg effect in HCC cells

**Fig. 2:**
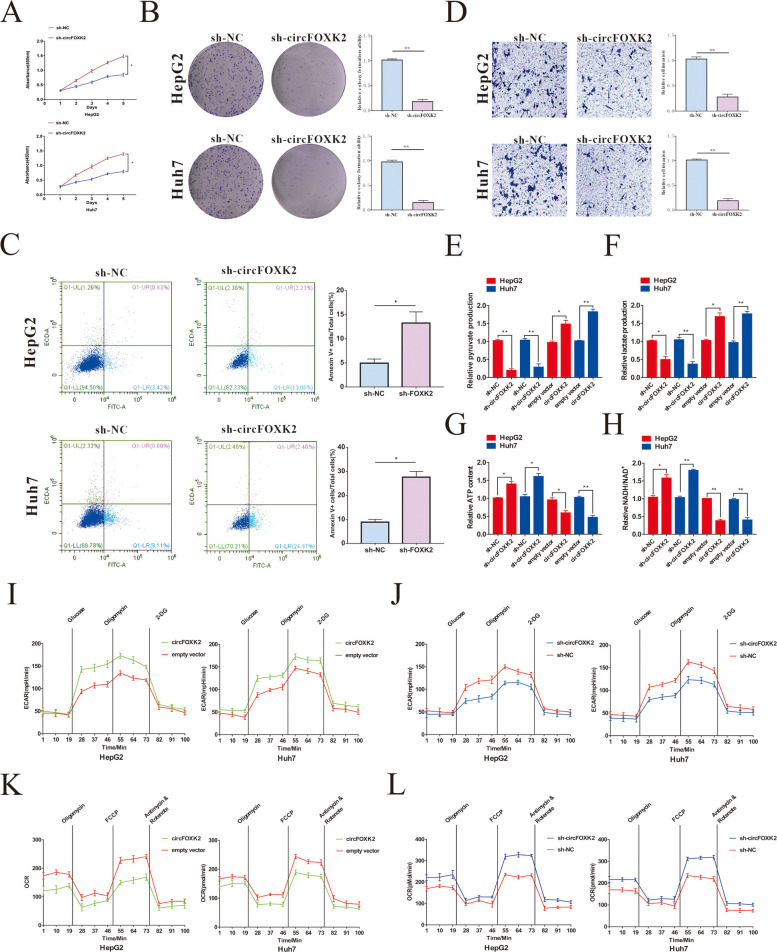
The effect of circFOXK2 on the ability of the progression and the Warburg effect of HCC cells. **A** Cell proliferation potentials in HepG2 and Huh 7 cells were assessed by CCK8 assay at day 1, 2, 3, 4 and 5 after respectively transfected sh-NC or sh-circFOXK2. **B** Representative images of colony formation of HCC cells by staining with crystal violet after knockdown of circFOXK2. Statistical analysis of colony formation assay. **C** Flow cytometry analysis was performed to detect the apoptotic rate of circFOXK2 silenced HepG2 and Huh7 cells. Statistical analysis of apoptosis assay. **D** Representative images of Transwell results of HCC cells after silencing of circFOXK2. Statistical analysis of Transwell assay. **E**–**G** Alterations in pyruvate production, lactate production and ATP production levels were detected after gain- or loss-expression of circFOXK2 of HepG2 and Huh7 cells. **H** The NADH/NAD + ratio was measured after transfection of HepG2 and Huh7 cells with sh-circFOXK2 or overexpression circFOXK2 vector. **I**-**J** The ECAR was measured after transfection of HepG2 and Huh7 cells with sh-circFOXK2 or overexpression circFOXK2 vector. **K**-**L** The OCR was detected after transfection of HepG2 and Huh7 cells with sh-circFOXK2 or overexpression circFOXK2 vector. Data were represented as means ± SD with at least three independent experiments. *p < 0.05, **p < 0.01, ***p < 0.001

To probe the biological functions of circFOXK2 in HCC, shRNA or a circFOXK2 overexpression vector was transfected into different HCC cell lines to conduct loss-and gain-to-function experiments. Stable cell lines (HepG2 and Huh7) of circFOXK2 knockdown were established by transfecting with shRNA-circFOXK2, while circFOXK2 overexpression cell lines were transfected with circFOXK2 overexpression vector (Supplemental Fig. 1A). As expected, the CCK8 and colony formation assays found that downregulation of circFOXK2 significantly suppressed the proliferation of HCC cells while enhancing their apoptotic rate, and overexpressing circFOXK2 yielded the opposite results (Fig. 2A-C and Supplemental Fig. 1B-D). In addition, we investigated the role of circFOXK2 in the invasion ability of HCC cells by conducting Transwell assays and wound healing assays. The results showed that knockdown of circFOXK2 expression remarkably suppressed HCC cell invasion, while overexpression of circFOXK2 potentiated HCC cell invasion (Fig. 2D and Supplemental Fig. 1E and F).

There is ample literature indicating that FOXK2 plays a critical role in the Warburg effect and is important for regulating the growth and metastasis of HCC. Therefore, we speculated that the protumor activity of circFOXK2 in HCC cells involves modulation of the Warburg effect. To verify this hypothesis, we established the stable HCC lines of circFOXK2 overexpression or knockdown and compared the critical cellular bioenergetics and metabolic parameters between these cell lines and the NC group (Supplemental Fig. 1A and B). As shown in Fig. 2E-F, compared with the NC group, significantly high pyruvate production and lactate accumulation in HCC cells were observed after circFOXK2 overexpression, whereas knockdown of circFOXK2 yielded the opposite results. Moreover, downregulated circFOXK2 significantly increased ATP formation from OXPHOS, which was reversed by circFOXK2 overexpression (Fig. 2G). The NADH/NAD + ratio was detected to further confirm the change of redox reaction after different treatments, and the results showed that compared with the NC group, knockdown of circFOXK2 significantly increased the ratio of NADH/NAD + , and this phenomenon could be reversed after transfection with circFOXK2 overexpressing plasmid (Fig. 2H). In addition, circFOXK2 overexpression enhanced the ECAR and impaired the oxygen consumption of HCC cells (Fig. 2I and J). In contrast, silencing circFOXK2 expression reduced the glycolytic capacity and enhanced the OCR (Fig. 2K and L). Taken together, these findings indicate the pro-oncogenic role of circFOXK2 in HCC and upregulated circFOXK2 could enhance the Warburg effect in HCC cells.

### CircFOXK2 encoded a 142 amino acid (aa) novel protein, FOXK2-142aa

**Fig. 3:**
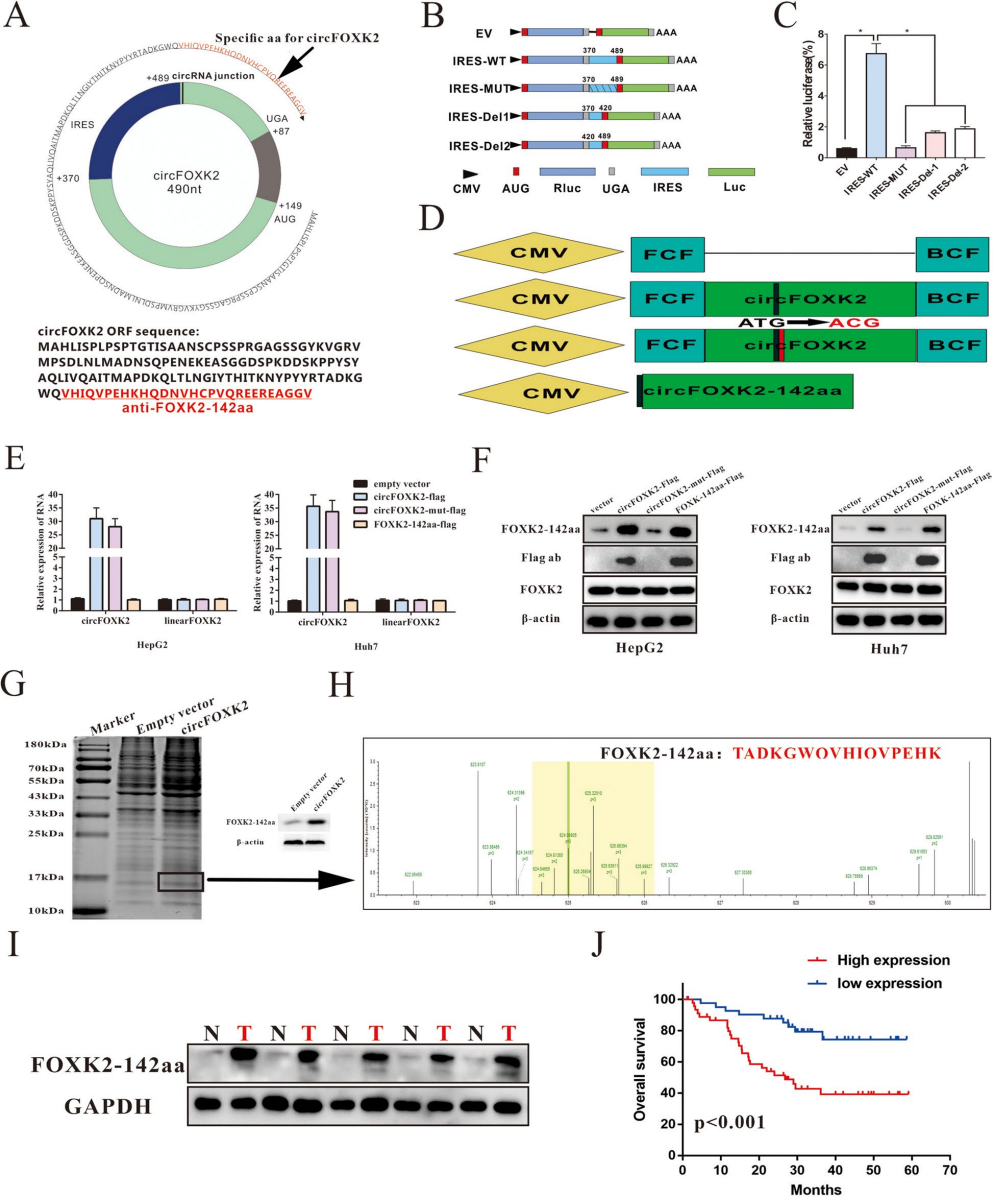
Verification of the encoding ability of circFOXK2. **A** circFOXK2 containing a putative ORF (upper panel). Illustration of the sequences of the putative ORF in circFOXK2 (lower panel). **B** The putative IRES activity of circFOXK2 was detected. The IRES sequences of circFOXK2 or its diverse truncations were inserted between Rluc and Luc reporter genes containing independent start and termination codons. **C** The luciferase activity of Rluc/Luc in the vectors mentioned above was measured. **D** Four vectors were established, including an empty vector, circFOXK2-flag (flag-tagged circFOXK2 sequence was carried by a CMV-induced expression vector), circFOXK2-fla-mut (flag-tagged circFOXK2 sequence with initial codon mutant, ATG → ACG, was carried by a CMV-induced expression vector) and FOXK2-142aa (flag-tagged FOXK2-142aa sequence was carried by a CMV-induced expression vector). FCF and BCF are presented as sequences that could circularize the circRNA sequence. **E** qRT-PCR was carried out to detect the relative RNA expression of circFOXK2 and linear FOXK2. **F** Western blot assay was performed to analyze the protein expression of flag-tagged FOXK2-142aa and FOXK2-142aa. **G** Left: Total proteins from the indicated cells were separated via SDS-PAGE, and the protein bands near 15 kDa were collected. FOXK2-142aa overexpression was verified by immunoblotting. (H) Right: LC–MS/MS was performed, and the FOXK2-142aa junction-specific peptide (TADKGWQVHIQVPEHK) was identified. **I** The expression of FOXK2-142aa was detected in HCC tissues and the counterpart ANTs. **J** The OS analysis was performed based on FOXK2-142aa expression in 92 HCC patients. Data were represented as means ± SD with at least three independent experiments. *p < 0.05, **p < 0.01, ***p < 0.001

In recent years, the protein-coding capacity of an increasing number of circRNA has gradually been brought to light. According to the original annotation from the circRNADb database, a 142 amino-acid (aa) protein is encoded by circFOXK2 (hsa_circ_0000817) which contains a 429-nt open reading frame (ORF) (Fig. 3A). Bioinformatics analysis showed that human circFOXK2 has a putative sequence of AUG in ORF that initiates the translation of a novel protein (Fig. 3A and Supplemental Fig. 2). Thus, the ORF of circFOXK2 was speculated to encode a putative 142 aa protein with the unique sequence of aa termed "FOXK2-142aa". Growing evidence demonstrates that the internal ribosome entry site (IRES) initiates the cap-independent translation process, which is a critical mechanism for circFOXK2 translation [30]. Thus, we conducted a dual-luciferase assay to verify the predictive effect of circFOXK2. As shown in Fig. 3B and C, the Luc/Rluc activity in circFOXK2 with full-length IRES was significantly higher than in the mutated or truncated IRES, whereas empty vectors did not stimulate Luc activation. In addition, to distinguish the role of FOXK2-142aa from circFOXK2, we established four flag-labeled vectors, including an empty vector, circFOXK2-flag, circFOXK2-flag-mut and FOXK2-142aa-flag (Fig. 3D). Among these, circFOXK2-flag represented a CMV-induced expression vector containing flag-labeled circFOXK2 sequence; circFOXK2-flag-mut represented a CMV-induced expression vector containing flag-labeled circFOXK2 sequence with start codon mutant (ATG → ACG), and FOXK2-142aa-flag represented a CMV-induced expression vector with the flag-labeled FOXK2-142aa sequence. Compared with the empty vector, the upregulated expression of circFOXK2 was investigated in HCC cells by transfecting with circFOXK2-flag vector or circFOXK2-flag-mut vector, while transfecting with FOXK2-142aa vector did not induce any change (Fig. 3E). Besides, these four vectors did not alter the mRNA expression of linear FOXK2 (Fig. 3E). Western blot provided preliminary evidence of the protein-encoding role of circFOXK2, which translated into FOXK2-142aa protein expression. In this regard, FOXK2 protein expression was not affected after transfection with the four vectors in Huh7 and HepG2 cells (Fig. 3F). Moreover, the expression of the flag was only observed in the cells transfected with circFOXK2-flag or FOXK2-142aa-flag, but not in the vector group or the circFOXK2-flag-mut group (Fig. 3F). Additionally, we prepared the specific antibody targeting FOXK2-142aa to verify this phenomenon and found that transfection with circFOXK2-flag or FOXK2-142aa-flag induced the high expression of FOXK2-142aa, and FOXK2-142aa could still be detected after transfection with an empty vector or circFOXK2-flag-mut, which indicated the presence of endogenous FOXK2-142aa (Fig. 3F). Furthermore, we separated the total cell lysate after transfecting circFOXK2 into HEK-293 T cells and then performed mass spectrometry analysis. The 15 kDa protein band obtained during SDS-PAGE was cut and underwent LC–MS/MS (Fig. 3G). The FOXK2-142aa encoded by circFOXK2 was confirmed, and the unique aa sequence of FOXK2-142aa, "TADKGWQVHIQVPEHK", was identified (Fig. 3H). Next, we used this specific antibody to detect the expression of FOXK2-142aa in clinical specimens. As expected, FOXK2-142aa expression was significantly higher in the HCC tissues than in the adjacent liver tissues (Fig. 3I). Finally, higher expression of FOXK2-142aa correlated with a worse OS of HCC patients (Fig. 3J). Collectively, these findings suggest that circFOKX2 is an encoding circRNA that could translate into the protein FOXK2-142aa in HCC.

### FOXK2-142aa interacted with LDHA to modulate its activity for affecting tumor promotion and the Warburg effect in HCC cells

**Fig. 4:**
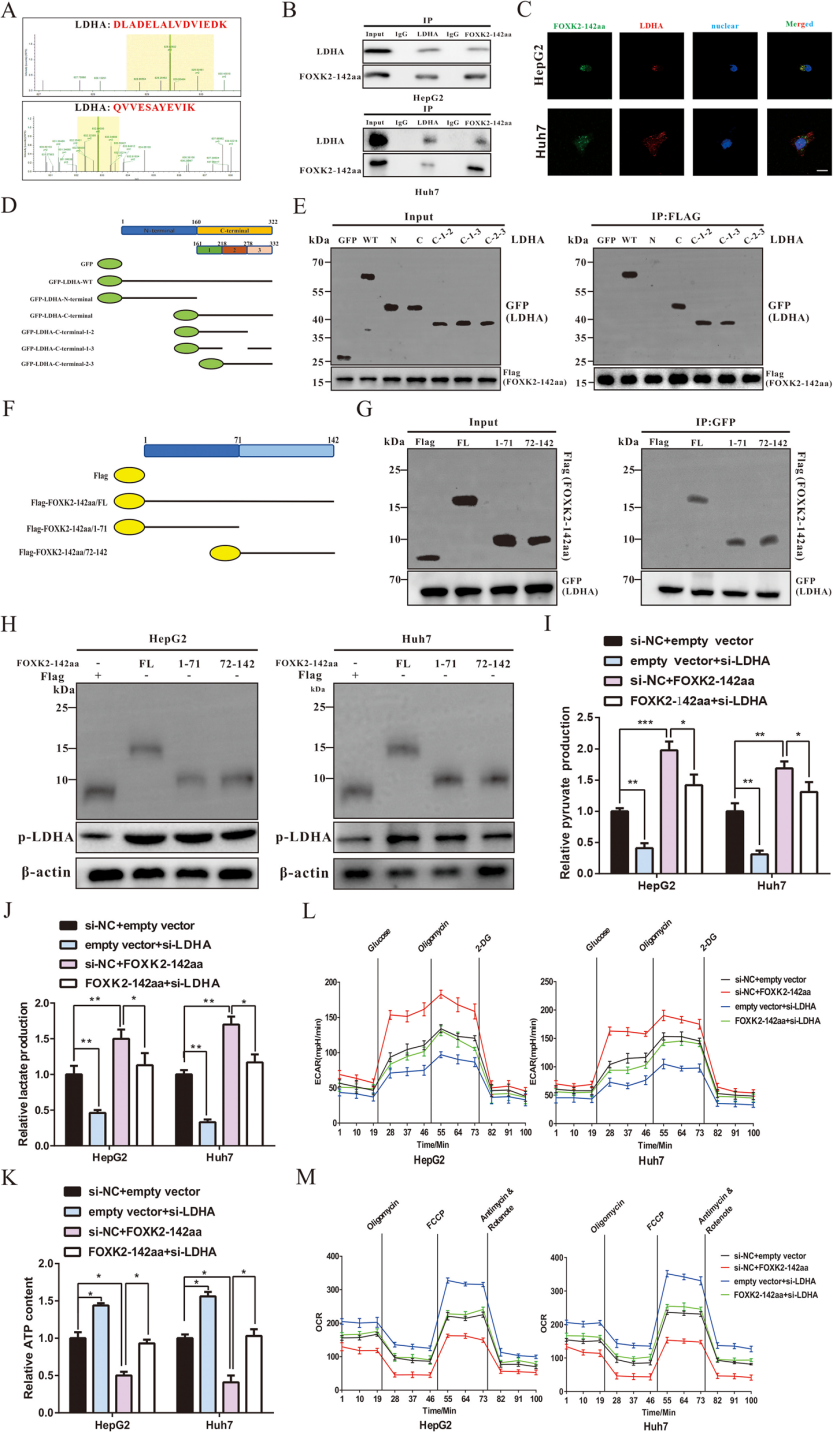
FOXK2-142aa interacts with LDHA and regulates its phosphorylation at the Tyr10 site in HCC. **A** The peptides of LDHA were identified by MS assay. **B** Co-IP with antibody against LDHA and FOXK2-142aa followed by Western blotting respectively using FOXK2-142aa and LDHA antibodies. **C** circFOXK2 overexpression plasmid was transfected into HepG2 and Huh7 cells, and immunofluorescence was conducted using anti-LDHA (fluorescent red) and anti-Flag (fluorescent green) antibodies. The nucleus was investigated by DAPI staining (fluorescent blue). (Bar = 2 μm). **D** Schematic diagrams showed the domain structure of LDHA and GFP-tagged LDHA mutants. **E** HEK-293 T cells were transfected with Flag-tagged FOXK2-142aa and GFP-tagged full-length or LDHA fragments, followed by performing an IP assay with an anti-Flag antibody. **F** Schematic of the wild-type FOXK2-142aa and its truncation mutants. **G** HEK-293 T cells were transfected with Flag-tagged FOXK2-142aa mutants and GFP-tagged LDHA, followed by conducting an IP assay with an anti-GFP antibody. **H** Western blot assay was performed to detect the level of phosphorylated LDHA in cells after transfected with wild-type FOXK2-142aa ORF or truncation mutants. **I**-**K** Alterations in pyruvate production, lactate production and ATP production levels were analyzed. **L**-**M** The ECAR and OCR in HepG2 and Huh7 cells were measured. Data were represented as means ± SD with at least three independent experiments. *p < 0.05, **p < 0.01, ***p < 0.001

To further investigate the function of FOXK2-142aa and its potential regulatory mechanism, we performed a co-IP assay and mass spectrum analysis to identify the putative interaction candidates of FOXK2-142aa. FOXK2-142aa-interacting proteins were analyzed by the Proteome Discover software (Thermo Fisher Scientific). As shown in Fig. 4A and Supplemental Fig. 3A, we found that the most abundant protein was lactate dehydrogenase A (LDHA), and FOXK2-142aa could bind to the peptides of LDHA, speculating that LDHA is the potential target of FOXK2-142aa. The interaction between FOXK2-142aa and LDHA was confirmed by the Western blot assay (Fig. 4B). In addition, after a circFOXK2 overexpression plasmid was transfected into HCC cells, we carried out immunofluorescence staining to further visualize the colocalization of FOXK2-142aa with LDHA in the cytoplasm of HCC cells (Fig. 4C). LDHA is well-recognized as an important enzyme contributing to the Warburg effect. Current evidence suggests that LDHA activity tightly depends on its phosphorylation status [31]. Upon phosphorylation at the residue (Tyr10) of LDHA, it promotes the conversion of NADH to NAD + , the generation of pyruvate and lactate, and is directly associated with the activities of several oncogenes to induce cancer progression [32]. Therefore, the four vectors mentioned above without flag-tag were transfected into HCC cells, and the phosphorylation level of LDHA was detected. It was found that transfection with the circFOXK2 or FOXK2-142aa vector significantly enhanced the phosphorylation at the Tyr10 site of LDHA, unlike the empty vector and circFOXK2-mut, suggesting that FOXK2-142aa interacted with LDHA to activate its Tyr10 (phosphorylation) in HCC cells (Supplemental Fig. 3B).

Moreover, we produced a series of Flag-tagged LDHA deletion mutants to delineate the domains of LDHA responsible for its interaction with FOXK2-142aa (Fig. 4D). Co-IP and Western blot assays showed that FOXK2-142aa preferred interacting with the C-terminal subunit of LDHA rather than its N-terminal domain (Supplemental Fig. 3C). In this regard, the △161–218 region of the C-terminal was indispensable for the LDHA-FOXK2-142aa binding (Fig. 4E). In the reciprocal experiments, the regions of FOXK2-142aa involved in interacting with LDHA were additionally analyzed by conducting a co-IP assay with Flag-tagged truncation mutants of FOXK2-142aa (Fig. 4F). As shown in Fig. 4G, FOXK2-142aa (1–71) and FOXK2-142aa (72–142) could be pulled down by LDHA, suggesting the full length of FOXK2-142aa interacted with LDHA. In a further study, increased LDHA phosphorylation was observed by full-length FOXK2-142aa expression rather than two truncation mutants, indicating that full-length FOXK2-142aa was responsible for regulating the phosphorylation of LDHA (Fig. 4H).

To further detect whether the biological effect of FOXK2-142aa on HCC progression was associated with LDHA, two vectors and one siRNA, including an empty vector, a FOXK2-142aa overexpression vector, and a si-LDHA, were transfected into HCC cells, respectively. Both CCK8 and colony formation assays revealed that LDHA knockdown suppressed the growth of HCC cells, while upregulation of FOXK2-142aa promoted HCC cell proliferation (Supplemental Fig. 3D-E). In addition, we transfected the FOXK2-142aa overexpression vector and si-LDHA into HCC cells and found that the exogenous knockdown of LDHA expression significantly weakened the pro-tumor effects of FOXK2-142aa (Supplemental Fig. 3D-E). The alterations in the Warburg effect in HCC cells were also investigated. Significantly higher pyruvate accumulation and lactate production were found after upregulating FOXK2-142aa expression, and these effects were reversed after further knockdown of LDHA (Fig. 4I and J). Moreover, the upregulation of FOXK2-142aa decreased the formation of ATP generated by OXPHOS; however, LDHA knockdown restored ATP formation (Fig. 4K). Consistent results were observed for the extracellular acidification rate. Combined transfection of si-LDHA and FOXK2-142aa overexpression vectors significantly reversed the FOXK2-142aa-induced decrease in OCR (Fig. 4L and M). These results corroborated that FOXK2-142aa plays an important role in affecting the progression and the Warburg effect of HCC cells, which closely correlates with the interaction of LDHA and stimulates its activation.

### In vivo potential of targeting FOXK2-142aa in HCC progression by upregulating LDHA activation

**Fig. 5:**
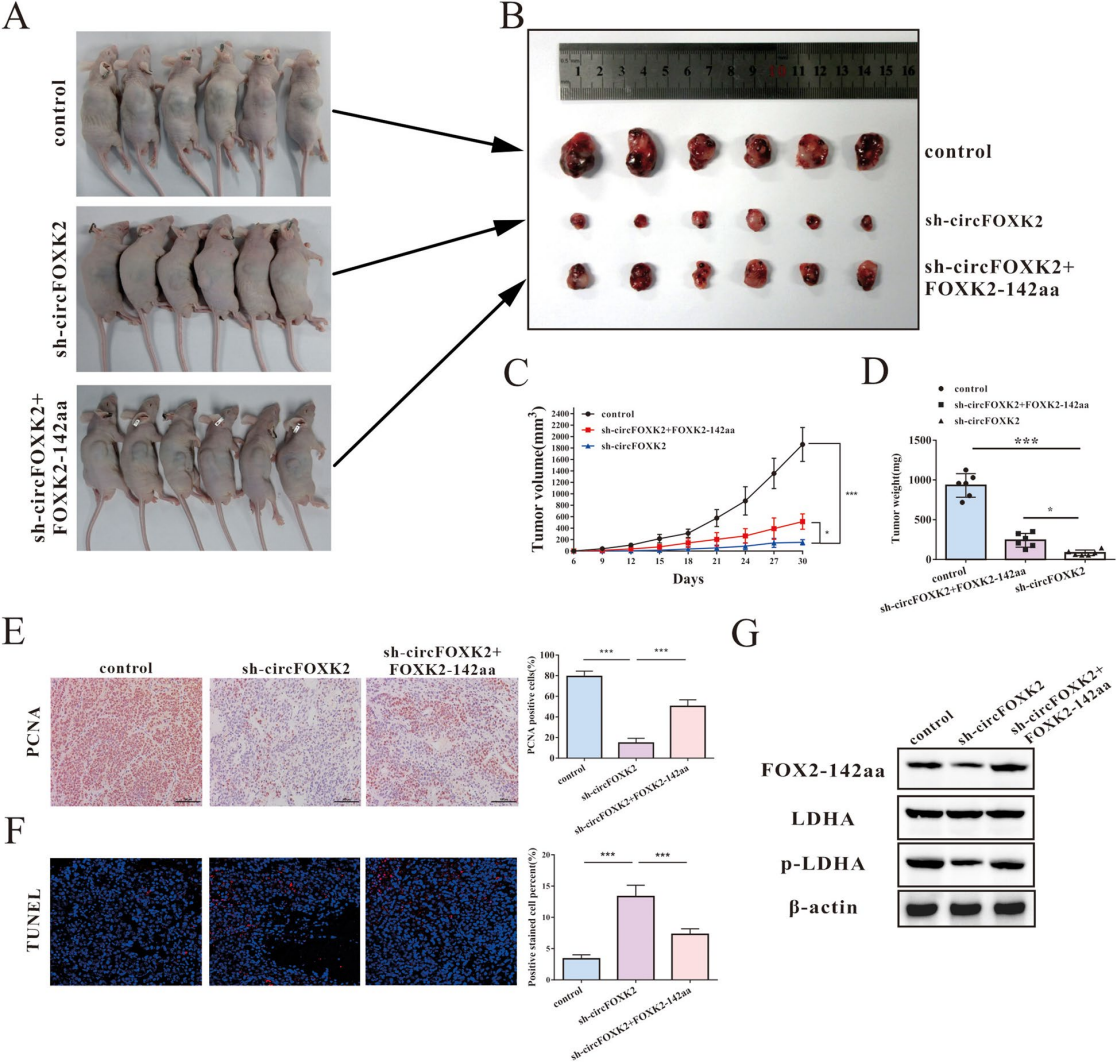
FOXK2-142aa positively correlates with the progression of HCC in vivo. **A**-**B** Huh7 cells stably receiving different treatments according to the group design (control, sh-circFOXK2 + FOXK2-142aa and sh-circFOXK2) were respectively used to subcutaneously inject into nude mice to establish xenograft tumors (*n* = 6 per group). A representative image of xenograft tumor formation in each group was obtained. **C** Analysis of the tumor volume among the three indicated groups measured three days. **D** The statistical analysis of the tumor weight among the three indicated groups. **E** Alterations in PCNA in xenograft tumors were investigated by IHC staining. (Bar = 20 μm). **F** The TUNEL assay was utilized to detect the apoptotic rate in xenograft tumors. **G** The expression of FOXK2-142aa, LDHA and p-LDHA in xenograft tumors in each group was detected by Western blot assay. The average intensity of the band was analyzed using β-actin as an internal control. Data were represented as means ± SD with at least three independent experiments. *p < 0.05, **p < 0.01, ***p < 0.001

To verify the in vitro results, we established xenograft tumor models by subcutaneous injection of HCC cells transfected with an empty vector, an shRNA lentiviral plasmid targeting circFOXK2 (sh-circFOXK2), and sh-circFOXK2 + a FOXK2-142aa overexpression plasmid (Fig. 5A and B). As shown in Figs. 5C and D, the tumors formed by circFOXK2-deficient HCC cells exhibited a significantly smaller volume and lower tumor weight than the NC group, which could be restored by further treatment with a FOXK2-142aa overexpression plasmid. PCNA and TUNEL staining confirmed the correlation between FOXK2-142aa and HCC tumorigenicity (Fig. 5E-F). Moreover, Western blot showed that compared with the NC group, FOXK2-142aa expression was reduced, and knockdown of circFOXK2 significantly decreased LDHA phosphorylation at Tyr10 in the tumor tissues while suppressing circFOXK2 and upregulating FOXK2-142aa expression could restore the phosphorylation levels (Fig. 5G). Finally, lung metastasis models were established to assess the metastatic capacity of HCC cells in each group. We found that the fluorescence intensities in the lungs were much weaker in the downregulated circFOXK2 expression group than in the NC group, and adding FOXK2-142aa overexpression plasmid reversed these findings, suggesting that circFOXK2 also promoted lung metastasis of HCC cells via encoding FOXK2-142aa (Supplemental Fig. 4A).

### CircFOXK2 additionally acts as a sponge targeting the tumor suppressor miR-484 in HCC

**Fig. 6:**
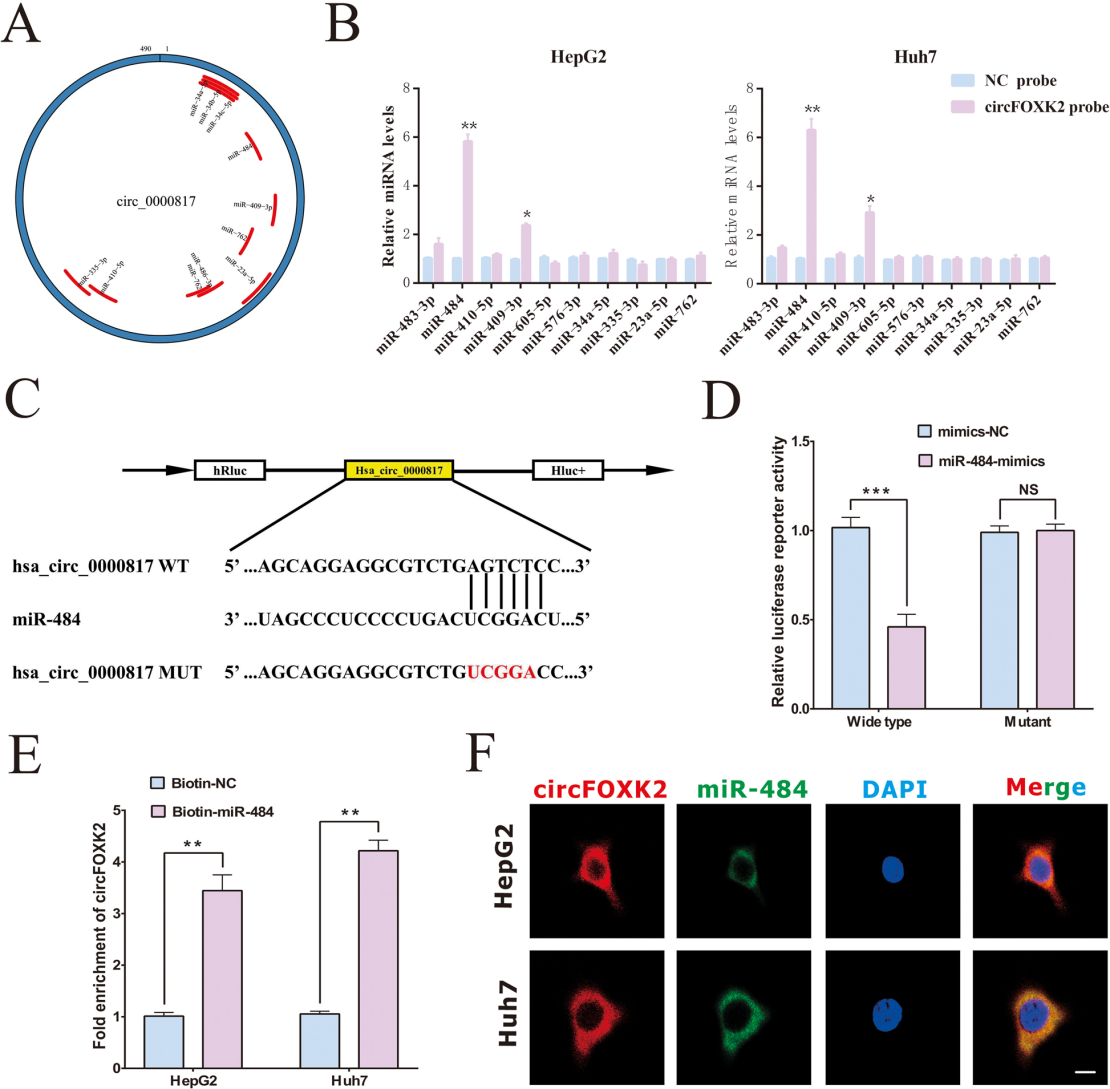
circFOXK2 acts as an miRNA sponge for miR-484 in HCC cells. **A** Map of circFOXK2 and its potential target miRNAs was depicted using Circlteractome. **B** qPCR analysis was conducted for the potential miRNAs pull-down by circFOXK2 in HepG2 and Huh7 cells. **C** Schematic illustrations exhibited the interactions of circFOXK2 with miR-484 by Arraystar's miRNA target prediction software. **D** The luciferase activity of wild-type and mutant circFOXK2 was detected by dual *Renilla* and firely luciferase reporter assays after co-transfected with either the miR-484 or control mimics in HepG2 and Huh7 cells. **E** RNA pulldown assays evaluated the circFOXK2 captured potential by biotinylated miR-484. **F** The locations of circFOXK2 (hsa_circ_0000817, red) and miR-484 (green) in HepG2 and Huh7 cells were detected using FISH. (magnification, 600 × ; scale bar, 100 μm). Data were represented as means ± SEM with at least three independent experiments. *p < 0.05, **p < 0.01, ***p < 0.001

It is well-acknowledged that circRNAs function as competing endogenous RNAs to regulate miRNA expression, resulting in the discharge of specific miRNA-targeted mRNAs [33]. In this study, we sought to explore the functions of circFOXK2. Ten putative miRNAs that could potentially bind to circFOXK2 (including miR-486-3p, miR-410-5p, miR-409-3p, miR-34c-5p, miR-34b-5p, miR-34a-5p, miR-335-3p, miR-23a-5p, miR-762, and miR-484) were predicted by an online database (RegRNA 2.0, http://regrna2.mbc.nctu.edu.tw/) (Fig. 6A). Subsequently, an RNA pull-down assay was conducted to assess whether circFOXK2 could directly interact with these candidate miRNAs. Biotin-labeled probes targeting circFOXK2 were constructed and used to conduct RNA pulldown in HCC cells, followed by qPCR assay to measure these ten miRNAs. Our results showed that miR-484 was the highest enriched miRNA in HepG2 and Huh7 (Fig. 6B). It has been demonstrated that miRNA could function as an RNA-induced silencing complex (RISC) component by interacting with Argonaute-2 (AGO2). Thus, we further conducted a RIP assay with anti-AGO2 and found that circFOXK2 and miR-484 were pulled down by the anti-AGO2 antibody (Supplemental Fig. 5A). In addition, the site of circFOXK2 binding miR-484 was predicted by RNAalifold (http://rna.tbi.univie.ac.at/), which was mutated to perform dual-luciferase reporter assays (Fig. 6C and Supplemental Fig. 5B). Both circFOXK2 wild-type (WT) luciferase reporter vector and circFOXK2 mutant luciferase reporter vector containing the sequence could not bind miR-484 were established and respectively co-transfected with miR-484 mimics into HepG2 and Huh7 cells. As shown in Fig. 6D, circFOXK2 WT luciferase reporter was significantly reduced by miR-484 mimics, while miR-484 did not affect the activity of circFOXK2 mutant luciferase reporter. Consistently, a pull-down assay with a biotin-labeled miR-484 probe found that circFOXK2 was captured by miR-484 (Fig. 6E). Finally, a FISH assay showed the colocalization of circFOXK2 and miR-484 in the cytoplasm of HepG2 and Huh7 cells (Fig. 6F). Taken together, these results indicated that circFOXK2 could directly bind to miR-484 for modulating the miR-484 expression in HCC cells.

### MiR-484 inhibits the progression and the Warburg effect of HCC cells via targeting the 3’-UTR of Fis1

**Fig. 7:**
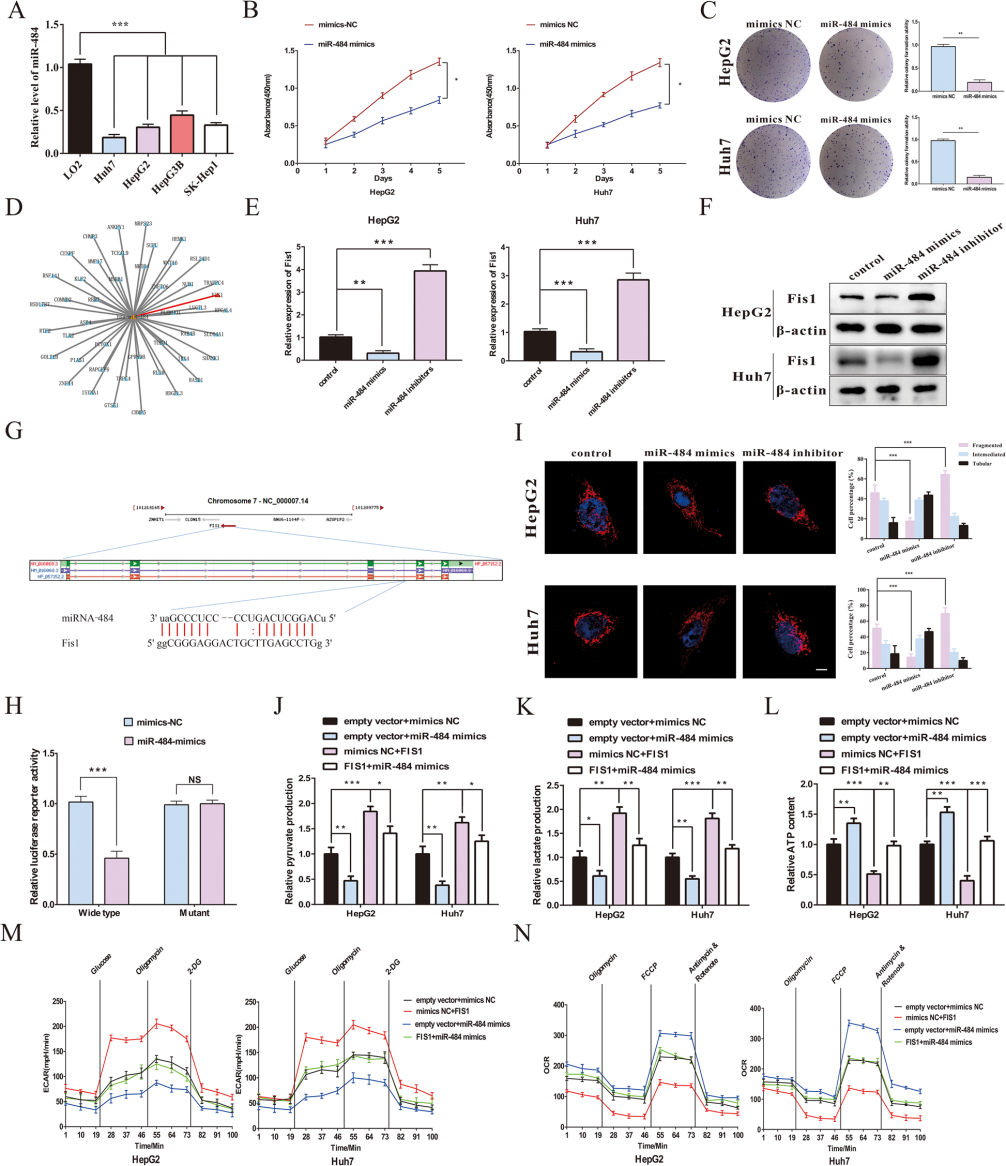
miR-484 regulates Fis1-related mitochondrial fission. **A** The expression of miR-484 in human HCC cell lines (Huh7, HepG2, Hep3B, and SK-Hep1) and normal hepatocyte line (LO2) were detected by RT-qPCR. **B** Cell proliferation potentials in HepG2 and Huh 7 cells were assessed by CCK8 assay at day 1, 2, 3, 4 and 5 after respectively transfected mimics-NC or miR-484 mimics. **C** Representative images of colony formation of HCC cells by staining with crystal violet after transfected mimics-NC or miR-484 mimics. Statistical analysis of colony formation assay. **D** The target genes of miR-484 predicted by miRWalk. **E** The effect of miR-484 mimics or miR-484 inhibitor on the mRNA expression of Fis1 in HCC cells was detected by qRT-PCR. **F** The protein expression of Fis1 was assessed in HCC cells after transfection with miR-484 mimics, miR-484 inhibitor or control mimics by Western blotting. **G** Schematic illustrations showed the alignment of miR-484 and Fis1. The red part was the mutagenesis nucleotides. **H** The luciferase activity of wild-type and mutant Fis1 was detected by dual *Renilla* and firely luciferase reporter assays after co-transfected with either the miR-484 or control mimics. **I** Mitochondrial morphology was evaluated using a confocal microscope after MitoTracker Green staining. The ratio of the morphological changes of mitochondria in per 100 cells in each group was analyzed according to the mitochondrial length. **J**-**L** Alterations in pyruvate production, lactate production and ATP production levels were detected in HepG2 and Huh7 cells. (M–N) The ECAR and OCR in HepG2 and Huh7 cells were measured. Data were represented as means ± SEM with at least three independent experiments. *p < 0.05, **p < 0.01, ***p < 0.001

It has been demonstrated that miR-484 plays a critical anti-cancer role in various tumors. Qiu et al. previously reported the effect of miR-484 on negatively regulating the progression of HCC cells [34]. In this study, we performed an RT-qPCR assay to detect the expression of miR-484 in several HCC cell lines and showed that miR-484 expression was downregulated in Huh7, HepG2, Hep3B, and SK-Hep1 cells compared with LO2 cells (Fig. 7A). Then, an miR-484 mimic was used to upregulate miR-484 expression, which could significantly reduce the proliferative potential of HCC cells, while treatment with a miR-484 inhibitor yielded the opposite results (Fig. 7B-C and Supplemental Fig. 6A-B).

It has been established that miRNAs can interact with the 3’-UTR of the target mRNAs to regulate their expression. Next, an online database, miRWalk, was applied to analyze the target genes that miR-484 could bind to (Fig. 7D). After miR-484 mimics and an inhibitor were transfected into HepG2 and Huh7 cell lines, the expression of Fis1 was upregulated and downregulated, respectively, at the mRNA and protein levels (Fig. 7E-F). We also constructed luciferase reporter plasmids containing wild-type Fis1 sequence or mutant Fis1 sequence and co-transfected them with miR-484 mimics to confirm whether Fis1 was a direct target of miR-484 in HCC cells (Fig. 7G). The results showed that miR-484 mimics reduced more than 50% of luciferase activity, whereas no effect on luciferase activity was observed after transfecting a vector comprising a mutant Fis1 sequence (Fig. 7H). Fis1 is a key factor affecting mitochondrial fission and subsequently regulating the Warburg effect. Therefore, we further detected the morphological changes of mitochondria in HCC cells. Compared with the control group, after treatment with a miR-484 inhibitor, more fragmented rod-shaped mitochondria were observed, while filamentous mitochondria were observed after transfecting with miR-484 mimics (Fig. 7I). Our findings indicated that the Warburg effect was weakened after transfecting with miR-484 mimics, as evidenced by the reduced levels of pyruvate and lactate and the increase of ATP generation, which were remarkably reversed by Fis1 overexpression (Fig. 7J-L). Similarly, the glycolysis ability in HCC cells, including the increase of ECAR level and the recession of OCR ability, inhibited by miR-484 mimics was also abolished after upregulated Fis1 expression (Fig. 7M-N). In summary, these findings demonstrated that miR-484 could inhibit the Warburg effect of HCC cells via regulating Fis1 expression and subsequently affecting mitochondrial dynamics.

### CircFOXK2 regulates Fis1-related mitochondrial fission and promotes the Warburg effect in HCC by acting as a ceRNA to sponge miR-484

**Fig. 8:**
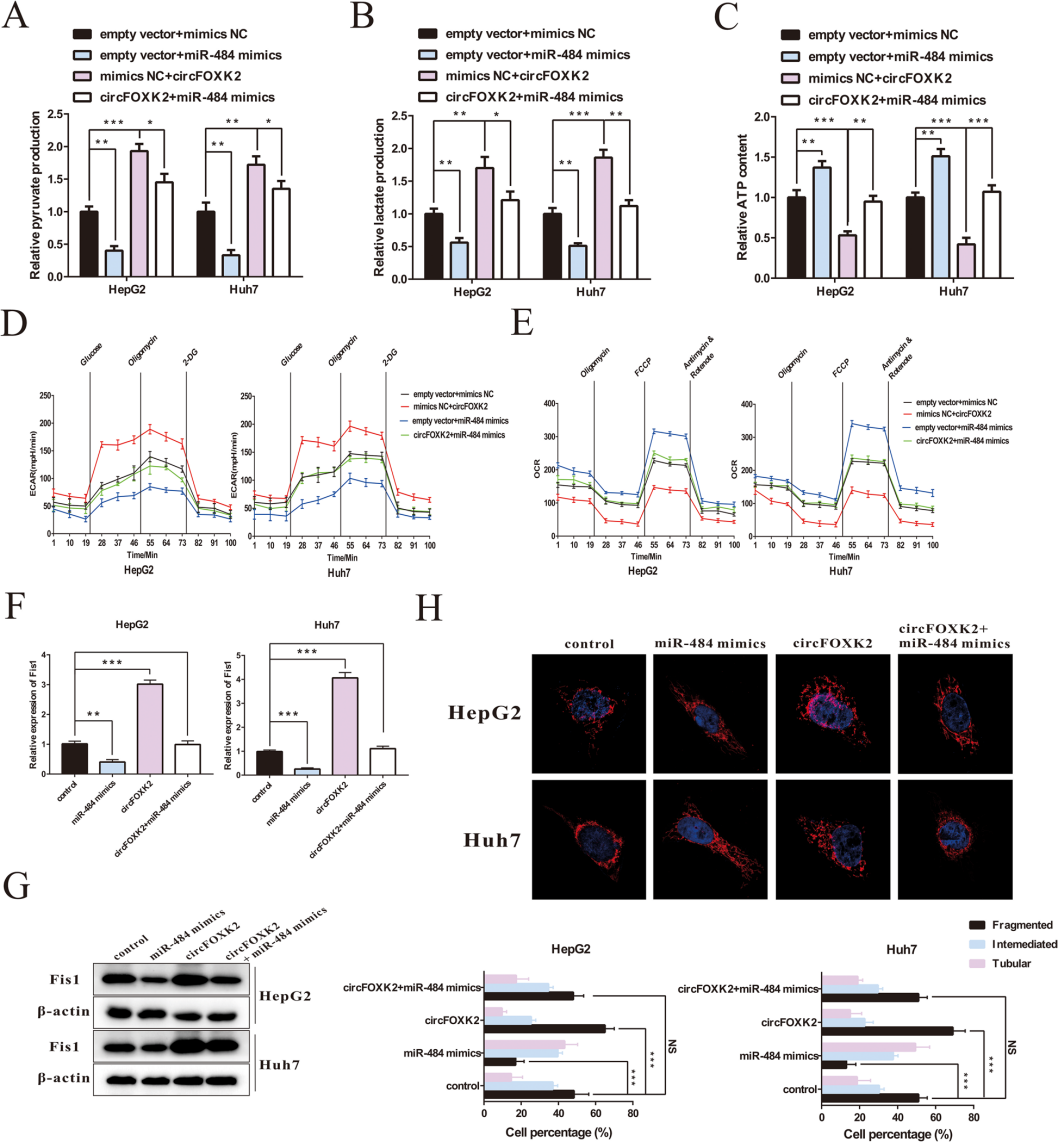
circFOXK2 regulates the Warburg effect in HCC cells via the axis of miR-484/Fis1-related mitochondrial fission. **A**-**C** Alterations in pyruvate production, lactate production and ATP production levels were detected in HepG2 and Huh7 cells. **D**-**E** The ECAR and OCR in HepG2 and Huh7 cells were measured. **F** The mRNA expression of Fis1 was assessed in HepG2 and Huh7 cells by qRT-PCR. **G** The protein expression of Fis1 was assessed in HepG2 and Huh7 cells by Western blotting. (H) Mitochondrial morphology was evaluated using a confocal microscope after MitoTracker Green staining. The ratio of the morphological changes of mitochondria per 100 cells in each group was analyzed according to the mitochondrial length. Data were represented as means ± SEM with at least three independent experiments. *p < 0.05, **p < 0.01, ***p < 0.001

Given that circFOXK2 could directly bind to miR-484 and miR-484 could inhibit the Warburg effect via regulating Fis1 expression, we carried out rescue experiments to detect whether circFOXK2 promotes the Warburg effect of HCC cells via not only encoding FOX2-142aa to regulate LDHA phosphorylation, but also acting as a ceRNA to sponge miR-484 followed by affecting Fis1-related mitochondrial fission. Upregulated miR-484 expression significantly reduced the levels of pyruvate and lactate in HCC cells; however, these effects could be rescued by combined overexpression of circFOXK2 (Fig. 8A and B). Moreover, increased ATP production induced by transfecting the miR-484 mimic was reversed after transfection of the circFOXK2 overexpression plasmids (Fig. 8C). In addition, the ECAR and OCR measurements showed that the glycolytic ability of HCC cells was significantly potentiated by overexpressing circFOXK2 and reversely decreased after further treatment with miR-484 mimics (Fig. 8D and E). The mRNA and protein expression of Fis1 was further detected. As shown in Figs. 8F and G, Fis1 expression could be enhanced by overexpressing circFOXK2, which could be blocked by introducing a miR-484 mimic. Moreover, we directly visualized the morphological changes of mitochondria in each group and found a more fragmented shape of mitochondria following the administration of a circFOXK2 overexpression vector, whereas mitochondria presented as filamentous configurations by combined application of a miR-484 mimic (Fig. 8H). Finally, we assessed the expression of Fis1 in the tumor specimens of the xenograft models. The results confirmed that Fis1 was significantly downregulated in the sh-circFOXK2 group compared with the control group (Supplemental Fig. 7A-B). Taken together, our findings substantiated that circFOXK2 upregulates Fis1 expression and subsequently induces mitochondrial fission to potentiate the Warburg effect in HCC cells by sponging miR-484 (Fig. 8).

**Fig. 9:**
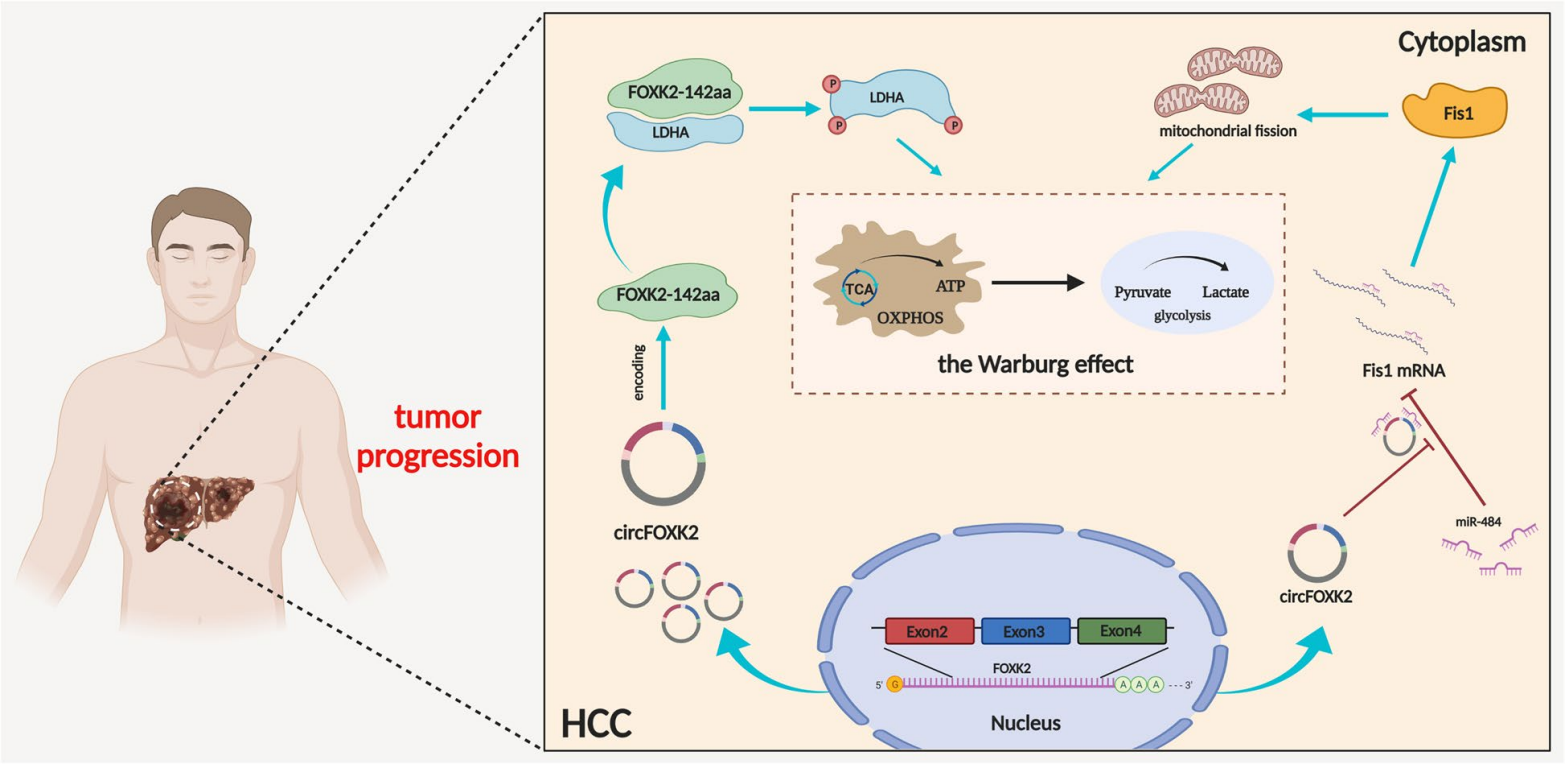
The effects of circFOXK2 on HCC. Schematic diagram illustrating the potential molecular mechanisms through which circFOXK2 regulates the Warburg effect and subsequently promotes HCC progression by not only translating into a novel protein, circFOXK2-142aa, to interact with LDHA and regulate its phosphorylation but also functions as a ceRNA to sponge miR484 and subsequently regulate Fis1 expression as well as induces mitochondrial fission

## Discussion

The Warburg effect plays a predominant role in cancer metabolism, promoting macromolecule biosynthesis and ATP generation and accounts for tumor progression and resistance to various therapeutics [13]. Based on the important role of the Warburg effect in HCC, in the present study, we identified a novel circRNA, circFOXK2 (hsa_circ_0000817), which was highly expressed in HCC tissues compared with their adjacent liver tissues and correlated with a poor prognosis. Next, we revealed its protein-encoding and miRNA sponging roles in mediating the Warburg effect.

FOXK2 is a critical transcription factor and a member of the forkhead box (FOX) family that contains a conserved helix DNA binding domain and regulates various biological processes, including the induction of aerobic glycolysis. The effect of FOXK2 on tumor progression remains controversial. FOXK2 can reportedly suppress tumor progression in breast cancer, renal carcinoma, and glioma, while contrasting findings have been reported for colorectal cancer and HCC [35–37]. Importantly, FOXK2 has been found to be upregulated in HCC tissues and promote HCC cell proliferation and migration via stimulating the PI3K/AKT signaling pathway [24].

Emerging evidence suggests that circRNAs are transcribed from precursor mRNA via back-splicing and participate in regulating various cancers. Recently, a circRNA derived from *FOXK2* (has_circ_0000816) has been found to promote the growth and metastasis of pancreatic ductal adenocarcinoma by sponging miR-942 and directly binding YBX1 and hnRNPK [38]. However, whether *FOXK2*-derived circRNA also affects the biological functions of HCC remains largely unknown. In this study, we assessed fourteen circRNAs derived from *FOXK2* and identified a novel circRNA (hsa_circ_0000817) that was significantly upregulated in HCC and plays an important role in promoting the progression and upregulating the Warburg effect of HCC. Indeed, it has been established that most circRNAs act as miRNA sponges or protein scaffolds. Interestingly, some circRNAs have recently been revealed to encode novel proteins or peptides that the translational procedure initiates in the IRES sequence and terminates in the novel stop codons [39]. In the present study, we demonstrated that circFOXK2 could encode a novel 142-aa protein, FOXK2-142aa. After treatment with a specific antibody targeting FOXK2-142aa, we showed that FOXK2-142aa was highly expressed in HCC and correlated with poor prognosis of HCC patients after radical hepatectomy. Mechanistically, the results from mass spectrum assay after co-IP revealed the correlation between FOXK2-142aa and LDHA. We found that FOXK2-142aa could interact with LDHA and activate its phosphorylation. It is well-recognized that LDHA activation induces pyruvate generation and promotes the catalysis of pyruvate to lactate, which is an essential checkpoint in driving the tricarboxylic acid (TCA) cycle anaplerosis to provide energy to HCC cells and potentiates their proliferation, invasion, and migration [31]. A previous study reported that hCINAP could interact with the C-terminal △219–278 of LDHA to modulate its phosphorylation [40]. In the present study, after LDHA was split into several sections, it was found that FOXK2-142aa could directly bind to the △161–218 C-terminal domain of LDHA and phosphorylated LDHA at Tyr10 site, thereby forming the FOXK2-142aa/LDHA signaling pathway to activate the Warburg effect in HCC.

Notably, both in vivo and in vitro experiments showed that blocking circFOXK2 encoding ability did not completely inhibit its pro-tumor role, suggesting the existence of other pathways to affect HCC progression. "miRNA sponging" is a circRNA function that represents a research hotspot in HCC [41]. As ceRNAs, many circRNAs interact with miRNAs via their miRNA response elements to regulate the biological effects of miRNAs, forming a complicated post-transcriptional modulatory network, a critical factor for cancer development [42]. CircMRPS35 could promote malignant progression and induce chemoresistance of HCC via sponging miR-148a [43]. Moreover, circRPN2 acts as a ceRNA for miR-183-5p to upregulate FOXO1 expression and suppress HCC progression [44]. Herein, based on bioinformatic analyses, RNA pull-down, and luciferase reporter assays, we identified that circFOXK2 contained miR-484 response elements and could specifically sponge to miR-484 that has been found to act as a tumor suppressor in various cancers, including cervical cancer and colorectal cancer [45, 46]. Li et al. reported that miR-484 suppresses pancreatic ductal adenocarcinoma (PDCA) growth via reducing Yes-associated protein (YAP) expression [47]. In the present study, we demonstrated that Fis1 and its downstream mitochondrial fission were targets of miR-484 in HCC. In this respect, it was found that miR-484 could mitigate Fis1 expression by binding to its 3’-UTR region. Fis1 is a receptor for dynamin-related protein 1 (Drp1) for the induction of mitochondrial fission that has been demonstrated to exhibit a key role in promoting the reprogramming of glucose metabolism and inducing the progression and anti-chemotherapy of cancer cells [48, 49]. Our rescue experiments found that circFOXK2 could regulate Fis1 expression and mitochondrial dynamics by interacting with miR-484. Notwithstanding that the present research revealed two regulatory mechanisms of circFOXK2 in HCC, more research is warranted to assess the existence of other potential signaling pathways.

## Conclusions

Overall, our findings provide compelling evidence that circFOXK2 is a hitherto undocumented oncogenic driver elevated in HCC tissues and correlated with HCC proliferation and invasion, which may serve as a prognostic biomarker in this patient population. Mechanistically, circFOXK2 encodes the protein FOXK2-142aa to interact with LDHA, phosphorylates Tyr10 and functions as a ceRNA to sponge miR-484 to regulate Fis1 expression and induce mitochondrial fission (Fig. 9). These effects finally enhance the Warburg effect in HCC cells. Our findings reveal a novel mechanism by which circRNAs promote the Warburg effect and regulate HCC pathogenesis. Excitingly, our findings may open up new avenues for the clinical application of circFOXK2 silencing to treat HCC patients.

## Supplementary Information

**Additional file 1:** **Supplemental Table 1.** The primer sequences used in this study. **Supplemental Table 2.** Relationship between circFOXK2 and clinical characteristics in HCC patients. **Supplemental Table 3.** Univariate and multivariate Cox-regression analysis of prognostic factors for HCC patients. **Supplemental Figure 1.** The effects of circFOXK2 on the ability of the progression of HCC cells. **Supplemental Figure 2.** The IRES site was predicted by circRNADb (http://reprod.njmu.edu.cn/cgi-bin/circrnadb/circRNADb.php). **Supplemental Figure 3.** FOXK2-142aa binds to LDHA to promote its phosphorylated activity. **Supplemental Figure 4.** FOX2-142aa positively correlates with the metastasis of HCC in vivo. **Supplemental Figure 5.** CircFOXK2 act as miRNA sponge for miR-484 in HCC cells. **Supplemental Figure 6.** The effect of miR-484 inhibitors on the progression of HCC cells. **Supplemental Figure 7.** circFOXK2 regulates Fis1 expression in HCC in vivo.   
