# Supplementary material for: CircFOXK2 promotes hepatocellular carcinoma progression and leads to a poor clinical prognosis via regulating the Warburg effect
Source: J Exp Clin Cancer Res. 2023 Mar 15;42:63. doi: 10.1186/s13046-023-02624-1 (PMC10018916; doi:10.1186/s13046-023-02624-1)
Supplement: Supplementary file 1 — Additional file 1: Supplemental Table 1. The primer sequences used in this study. Supplemental Table 2. Relationship between circFOXK2 and clinical characteristics in HCC patients. Supplemental Table 3. Univariate and multivariate Cox-regression analysis of prognostic factors for HCC patients. Supplemental Figure 1. The effects of circFOXK2 on the ability of the progression of HCC cells. Supplemental Figure 2. The IRES site was predicted by circRNADb (http://reprod.njmu.edu.cn/cgi-bin/circrnadb/circRNADb.php). Supplemental Figure 3. FOXK2-142aa binds to LDHA to promote its phosphorylated activity. Supplemental Figure 4. FOX2-142aa positively correlates with the metastasis of HCC in vivo. Supplemental Figure 5. CircFOXK2 act as miRNA sponge for miR-484 in HCC cells. Supplemental Figure 6. The effect of miR-484 inhibitors on the progression of HCC cells. Supplemental Figure 7. circFOXK2 regulates Fis1 expression in HCC in vivo. [file 13046_2023_2624_MOESM1_ESM.docx]

# Supporting information

## CircFOXK2 Promotes Hepatocellular Carcinoma Progression and Leads a Poor Clinical Prognosis via Regulating the Warburg Effect

Jun Zheng, Xijing Yan, Tongyu Lu, Wen Song, Yang Li, Jinliang Liang, Jiebin Zhang, Jianye Cai, Xin Sui, Jiaqi Xiao, Haitian Chen, Guihua Chen, Jia Yao, Kejing Zeng, Qi Zhang, Yubin Liu, Yang Yang, Kanghong Zheng, Zihao Pan

**Supplemental Table 1. The primer sequences used in this study**

| Primer name | Primer sequence (5’→3’) |
| --- | --- |
| Primers for real-time PCR |  |
| Circ_0000817 | F: AGGAAGCTTCAGGTGGAGAC |
|  | R: GGAACCTGAATGTGCACCTG |
| Circ_0000816 | F: TCTTCAGGGTACAAGGTGGG |
|  | R: AACCTGAATGTGCACCTTCG |
| Circ_0046423 | F: GCCCCTGACCATCAACATTC |
|  | R: ACGCGCGGCTGATGGTTC |
| Circ_0046425 | F: CTCACCCTGAACGGGATTTA |
|  | R: GGGAGCACGCGCGGCTGCCA |
| Circ_0046428 | F: GCCCCTGACCATCAACATTC |
|  | R: GGAACCTGAATGTGCACCTGAT |
| Circ_0046429 | F: CAGGGAAGTCAAAGGTGCAC |
|  | R: GAATGTTGATGGTCAGGGGC |
| Circ_0046430 | F: GTAGAGCCTATTCCCGCCAT |
|  | R: ATGTGCACCATTGTTCACCT |
| Circ_0046431 | F: GAAGTCAAAGCGCTGCAAAC |
|  | R: GTCTCCACCTGAAGCTTCCT |
| Circ_0046432 | F: CTGGGACCGCTCTCTTCTAG |
|  | R: CTTTGAATCATCCTGGGGCG |
| Circ_0046433 | F: GCGAACACGTACACTGTCTC |
|  | R: GGCGGCTTTGAATCATCCTTTG |
| Circ_0046434 | F: GTAGAGCCTATTCCCGCCAT |
|  | R: GGCGGCTTTGAATCATCCAT |
| Circ_0046435 | F: GGCCAGGTGAACAATGAATTC |
|  | R: CTAGAAGAGAGCGGTCCCAG |
| Circ_0046436 | F: CCAGGTGAACAATGGGTCAC |
|  | R: GACCACAGCTTGTCCAGAGA |
| Circ_0107802 | F: GGATGATTCAAAGCCGCCTT |
|  | R: CAGGAGTTTGCAGCGCTGCCA |
| liner FOXK2 | F: ATGGCTCCCGACAAACAGCT |
|  | R: TGGACAGGGCAGTGAACGTT |
| miR-484 | F: CGACGGATCCAAGCGCACCCTTCACTTC |
|  | R: GCTCGAATTCCGCTTCAAGGTTCCTTTCG |
| Fis1 | F: CTGGACTCATTGGACTGGCTGTG |
|  | R: AGGAAGGCGGTGGTGAGGATG |
| GAPDH | F: GGAGCGAGATCCCTCCAAAAT |
|  | R: GGCTGTTGTCATACTTCTCATGG |
| divergent GAPDH | F: GAAGGTGAAGGTCGAGTC |
|  | R: GAAGATGGTGATGGGATTTC |
| β-actin | F: CACCATTGGCAATGAGCGGTTC |
|  | R: AGGTCTTTGCGGATGTCCACGT |

| **Supplemental Table 2. Relationship between circFOXK2 and clinical characteristics in HCC patients** | | | | | |
| --- | --- | --- | --- | --- | --- |
|  | **Low expression** |  | **High expression** |  | P |
| **Gender** | N | % | N | % | 0.239 |
| Male | 32 | 69.57 | 37 | 80.43 |  |
| Female | 14 | 30.43 | 9 | 19.57 |  |
| **T stage** |  |  |  |  | 0.694 |
| T1 | 9 | 19.57 | 6 | 13.04 |  |
| T2 | 7 | 15.22 | 8 | 17.39 |  |
| T3 | 30 | 65.21 | 32 | 69.57 |  |
| **N stage** |  |  |  |  | 0.209 |
| N0 | 24 | 52.17 | 18 | 39.13 |  |
| N1 | 22 | 47.83 | 28 | 60.87 |  |
| **Differentiation** |  |  |  |  | 0.757 |
| Well | 13 | 28.26 | 12 | 26.09 |  |
| Moderate | 24 | 52.17 | 22 | 47.83 |  |
| Poor | 9 | 19.57 | 12 | 26.08 |  |

*P<0.05

| **Supplemental Table 3. Univariate and multivariate Cox-regression analysis of prognostic factors for HCC patients** | | | | | | | | |
| --- | --- | --- | --- | --- | --- | --- | --- | --- |
|  |  | Univariate analysis | |  |  | | Multivariate analysis |  |
|  | HR | 95% CI | | P | HR | | 95% CI | P |
| T stage |  |  | |  |  | |  |  |
| T1 | 1 |  | |  | 1 | |  |  |
| T2 | 10.03 | (1.23, 81.66) | | 0.031^*^ | 6.93 | | (0.85,56.88) | 0.071 |
| T3 | 8.32 | (1.13, 61.38) | | 0.038^*^ | 5.26 | | (0.70,39.51) | 0.107 |
| N stage |  |  |  | | |  |  |  |
| N0 | 1 |  | |  | | 1 |  |  |
| N1 | 3.20 | (1.49,6.89) | | 0.003^**^ | | 2.47 | (1.11,5.51) | 0.027^*^ |
| Gender |  |  | |  | |  |  |  |
| Male | 1 |  | |  | | 1 |  |  |
| Female | 0.18 | (0.04,0.74) | | 0.017^*^ | | 0.20 | (0.05,0.84) | 0.029^*^ |
| Differentiation |  |  | |  | |  |  |  |
| Well | 1 |  | |  | |  |  |  |
| Moderate | 1.37 | (0.63,2.99) | | 0.421 | | - | - | - |
| Poor | 0.28 | (0.08,1.03) | | 0.056 | | - | - | - |
| Expression of  CircFOXK2 |  |  | |  | |  |  |  |
| Low | 1 |  | |  | | 1 |  |  |
| High | 3.55 | (1.65,7.63) | | 0.001^***^ | | 2.87 | (1.31, 6.24) | 0.008^**^ |

*P<0.05, **P<0.01, ***P<0.001


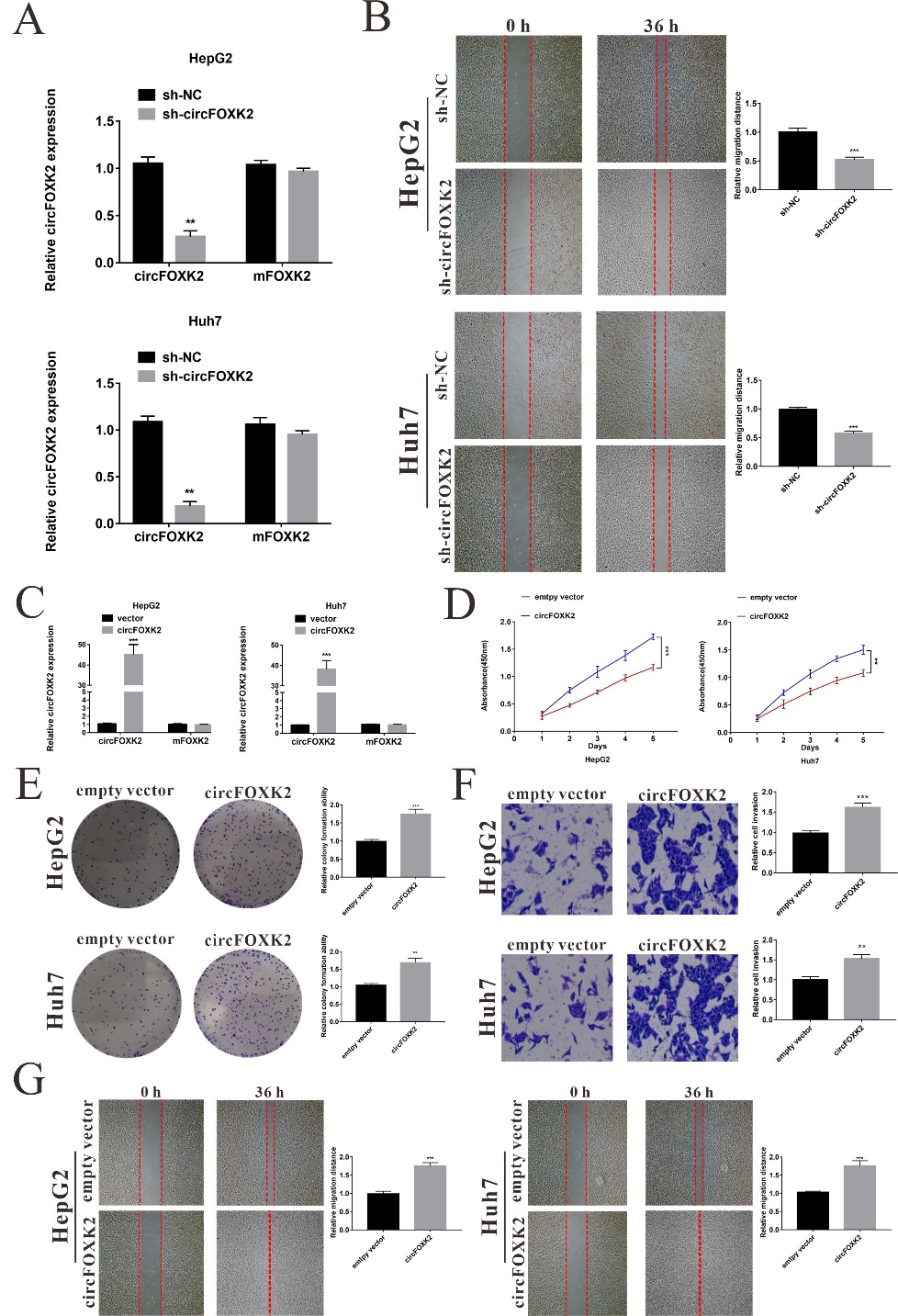


**Supplemental Figure 1.** The effects of circFOXK2 on the ability of the progression of HCC cells. (A) qRT-PCR assay was performed to confirm the expression of circFOXK2 in both HepG2 and Huh7 cells transfected with the shRNA targeting circFOXK2. (B) Representative images and quantitative analysis for the wound healing assay for HepG2 and Huh7 cells. (C) The expression of circFOXK2 in both HepG2 and Huh7 cells after treated a circFOXK2 overexpression vector. (D) Cell proliferation potentials in HepG2 and Huh 7 cells were assessed by CCK8 assay at day 1, 2, 3, 4 and 5 after respectively transfected empty vector or circFOXK2 overexpression vector. (E) Representative images of colony formation of HCC cells by staining with crystal violet after overexpressing circFOXK2. Statistical analysis of colony formation assay. (F) Representative images of Transwell results of HCC cells after overexpressing circFOXK2. Statistical analysis of Transwell assay. (G) Representative images and quantitative analysis for the wound healing assay for HepG2 and Huh7 cells. Data were represented as means ± SEM with at least three independent experiments. *p < 0.05, **p < 0.01, ***p < 0.001


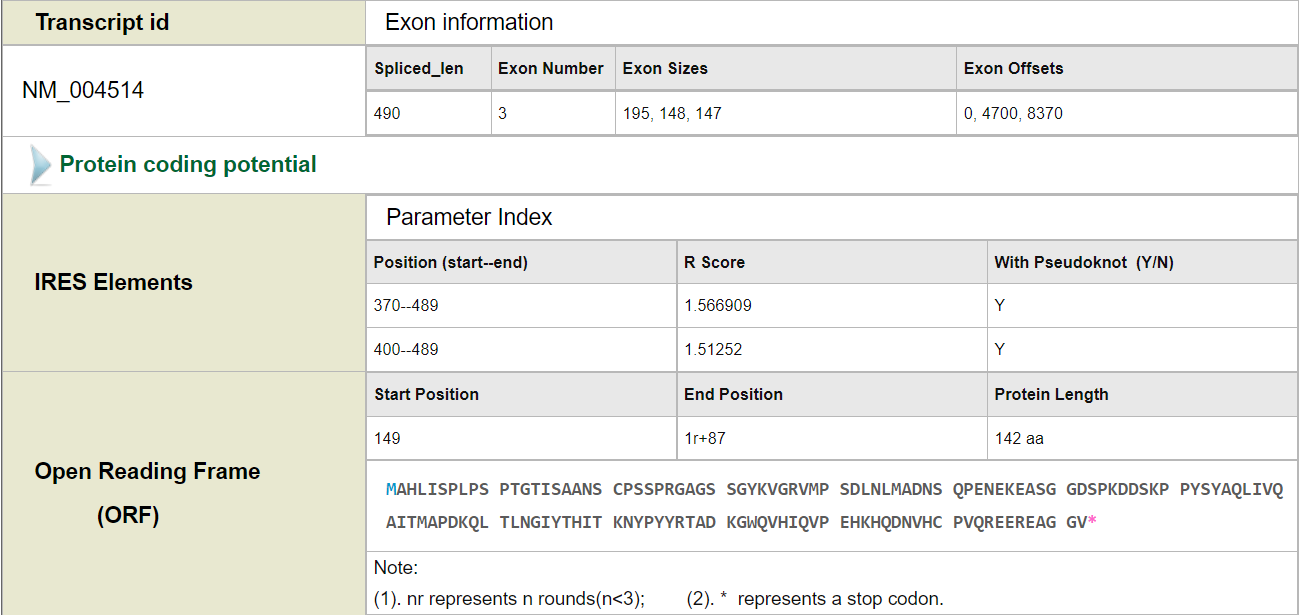


**Supplemental Figure 2.** The IRES site was predicted by circRNADb (http://reprod.njmu.edu.cn/cgi-bin/circrnadb/circRNADb.php).


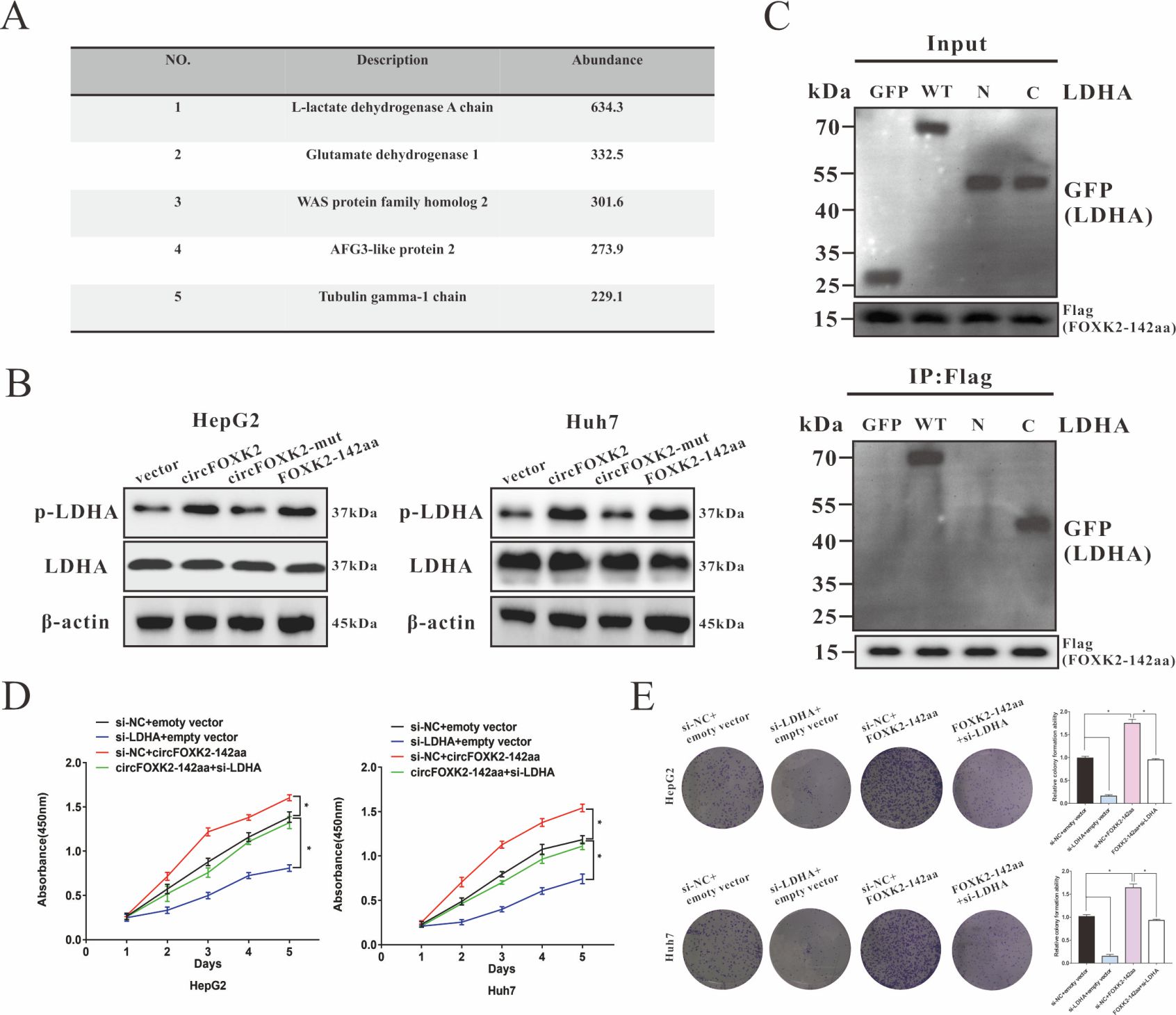


**Supplemental Figure 3.** FOXK2-142aa binds to LDHA to promote its phosphorylated activity. (A) The table presents mass spectrometric results identifying binding proteins of FOXK2-142aa. (B) The expressions of LDHA and p-LDHA in HepG2 and Huh7 cells after different treatments according to the group design were determined by Western blot. The average intensity of the band was analyzed using β-actin as an internal control. (C) HEK-293T cells were transfected with Flag-labeled FOXK2-142aa and GFP-tagged full-length or LDHA fragments, followed by performing immunoprecipitation (IP) by anti-Flag antibody. (D) Cell proliferation potentials in HepG2 and Huh7 cells were evaluated by CCK8 assay at day 1, 2, 3, 4 and 5 after respectively transfected an empty vector, si-LDHA, FOXK2-142aa overexpression plasmid or FOXK2-142aa overexpression plasmid + si-LDHA. (E) Representative images of colony formation of HCC cells by staining with crystal violet after respectively transfected an empty vector, si-LDHA, FOXK2-142aa overexpression plasmid or FOXK2-142aa overexpression plasmid + si-LDHA. Statistical analysis of colony formation assay. Data were represented as means ± SEM with at least three independent experiments. *p < 0.05, **p < 0.01, ***p < 0.001.


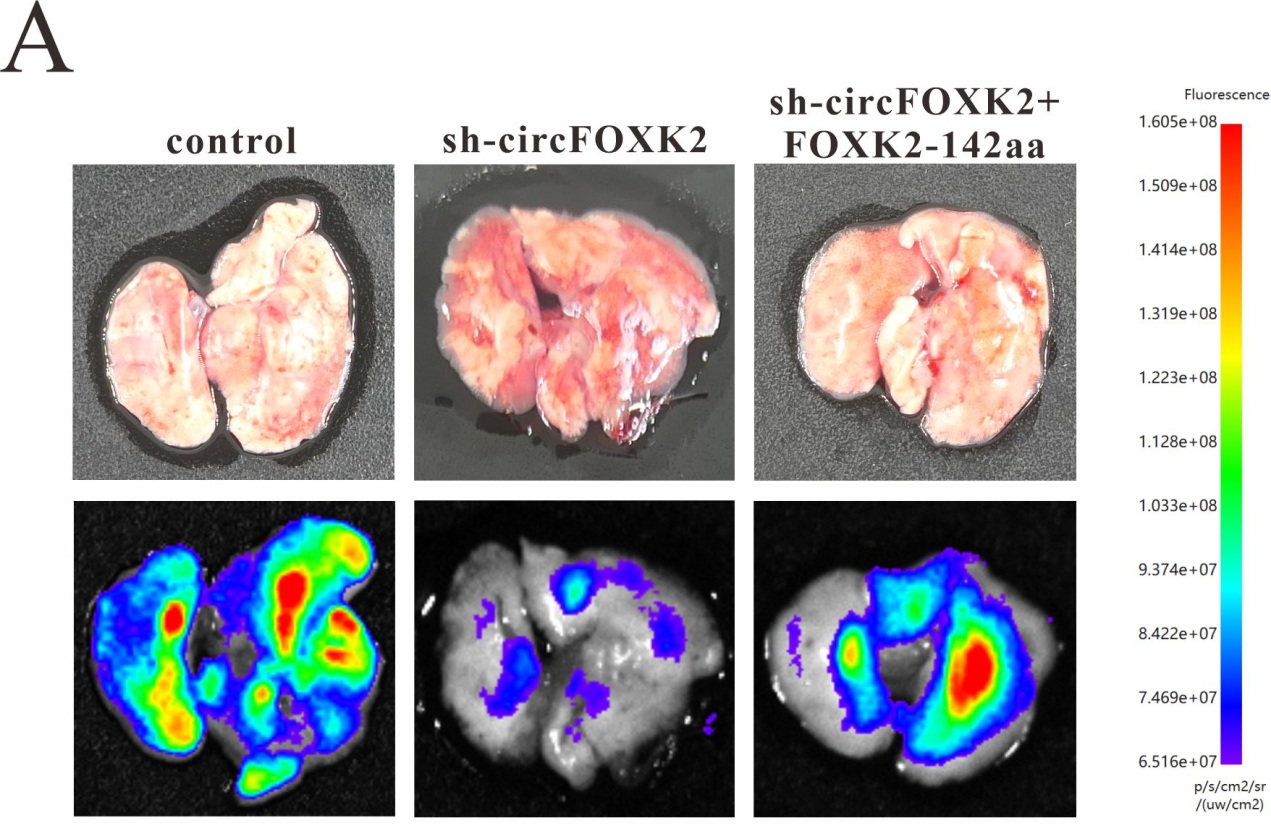


**Supplemental Figure 4. FOX2-142aa positively correlates with the metastasis of HCC in vivo.**

(A) Representative lungs (upper) and relative fluorescence (lower) images for each group after injection of indicated HCC cells through tail vein.


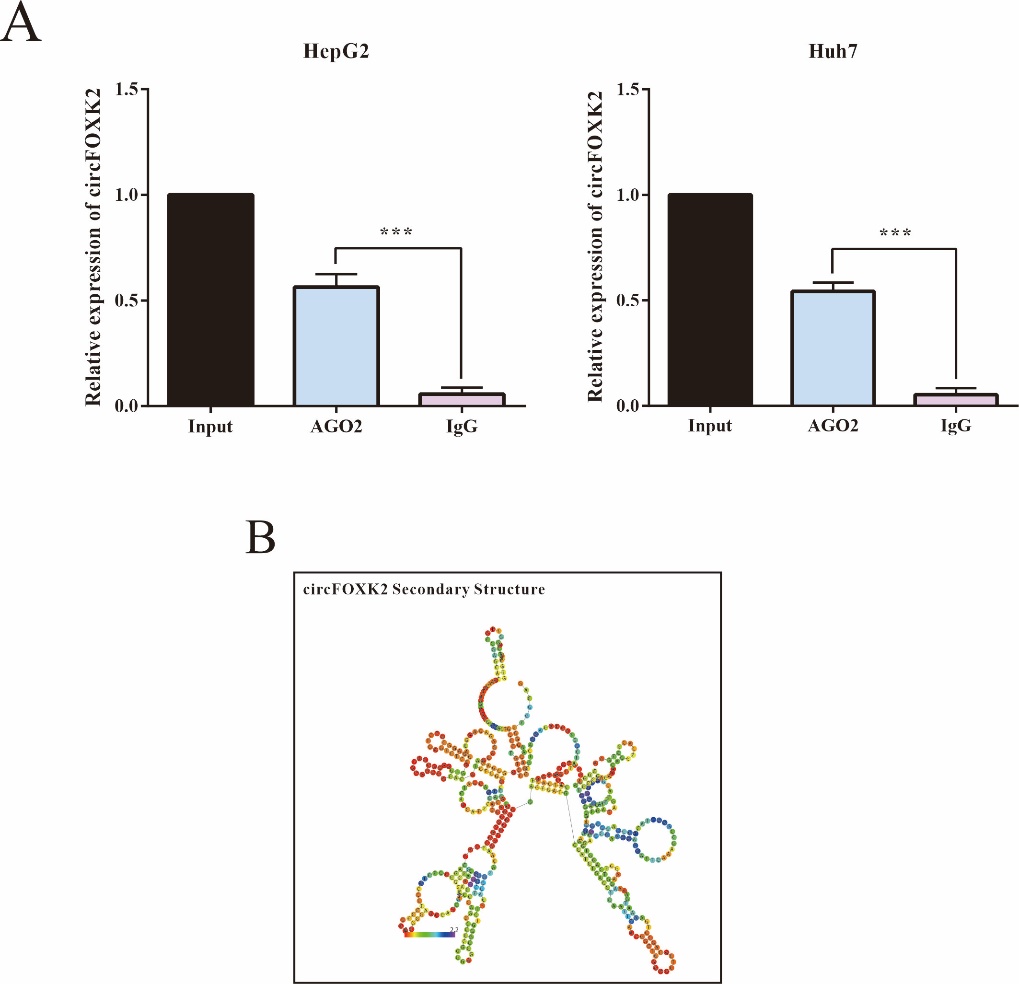


**Supplemental Figure 5.** CircFOXK2 act as miRNA sponge for miR-484 in HCC cells. (A) Anti-AGO2 RIP was conducted to detect circFOXK2 and miR-484 in HepG2 and Huh7 cells. (B) RNAalifold was used to predict the secondary structure of circFOXK2. Data were represented as means ± SEM with at least three independent experiments. *p < 0.05, **p < 0.01, ***p < 0.001.


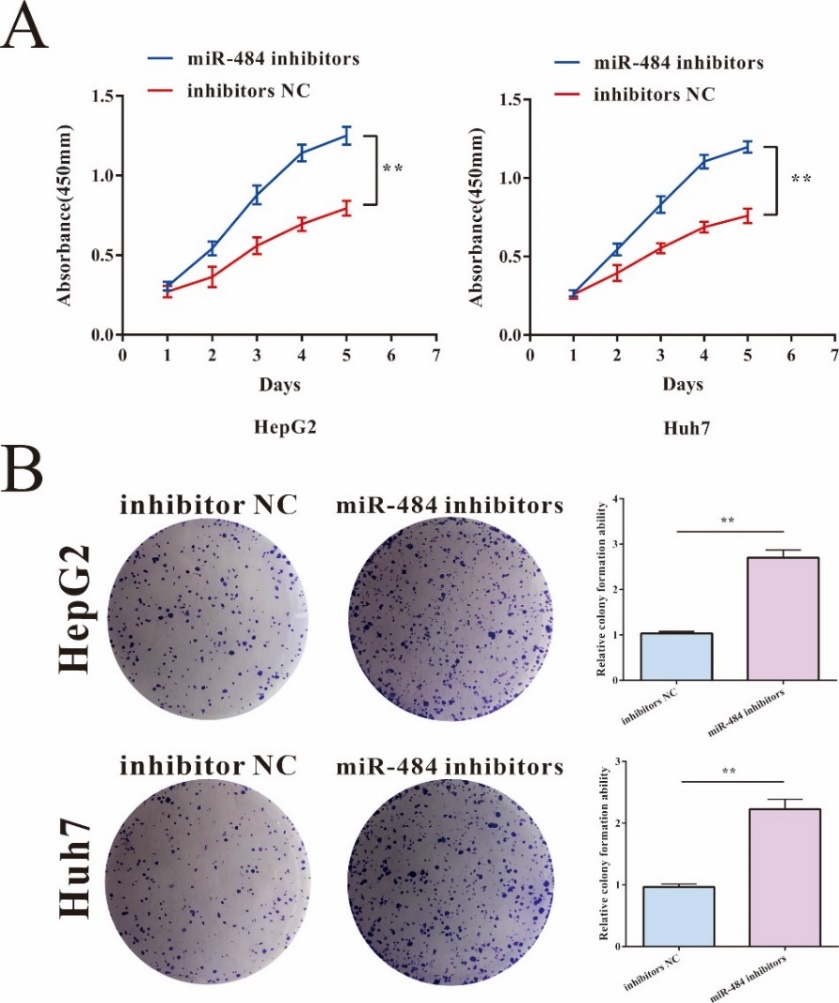


**Supplemental Figure 6.** The effect of miR-484 inhibitors on the progression of HCC cells. (A) Cell proliferation potentials in HepG2 and Huh7 cells were evaluated by CCK8 assay at day 1, 2, 3, 4 and 5 after respectively treated with miR-484 inhibitors or inhibitors NC. (B) Representative images of colony formation of HCC cells by staining with crystal violet after respectively treated with miR-484 inhibitors or inhibitors NC. Data were represented as means ± SEM with at least three independent experiments. *p < 0.05, **p < 0.01, ***p < 0.001.


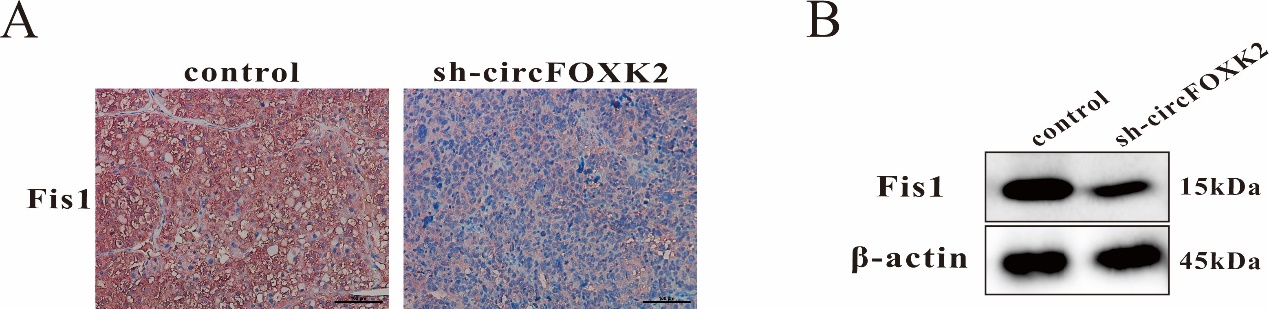


**Supplemental Figure 7.** circFOXK2 regulates Fis1 expression in HCC in vivo. (A) Alteration in Fis1 expression in xenograft tumors were investigated by IHC staining. (Bar= 20 μm). (J) The expression of Fis1 in xenograft tumors was detected by Western blot assay. The average intensity of the band was analyzed using β-acin as an internal control. Data were presented as means ± SEM (n=5 mice per group).
